# Multiplex Detection of Rare Mutations by Picoliter Droplet Based Digital PCR: Sensitivity and Specificity Considerations

**DOI:** 10.1371/journal.pone.0159094

**Published:** 2016-07-14

**Authors:** Eleonora Zonta, Fanny Garlan, Nicolas Pécuchet, Karla Perez-Toralla, Ouriel Caen, Coren Milbury, Audrey Didelot, Elizabeth Fabre, Hélène Blons, Pierre Laurent-Puig, Valérie Taly

**Affiliations:** 1 Université Sorbonne Paris Cité (USPC), INSERM UMR-S1147, CNRS SNC 5014, Centre Universitaire des Saints-Pères, Paris, France; 2 Medical oncology, Hôpital Européen Georges Pompidou (HEGP), Assistance Publique Hôpitaux de Paris (AP-HP), Paris, France; 3 Department of Biochemistry, Unit of pharmacogenetic and molecular oncology, Hôpital Européen Georges Pompidou (HEGP), Assistance Publique Hôpitaux de Paris (AP-HP), Paris, France; 4 RainDance Technologies, Billerica, Massachusetts, United States of America; University of Navarra, SPAIN

## Abstract

In cancer research, the accuracy of the technology used for biomarkers detection is remarkably important. In this context, digital PCR represents a highly sensitive and reproducible method that could serve as an appropriate tool for tumor mutational status analysis. In particular, droplet-based digital PCR approaches have been developed for detection of tumor-specific mutated alleles within plasmatic circulating DNA. Such an approach calls for the development and validation of a very significant quantity of assays, which can be extremely costly and time consuming. Herein, we evaluated assays for the detection and quantification of various mutations occurring in three genes often misregulated in cancers: the epidermal growth factor receptor (*EGFR*), the v-Ki-ras2 Kirsten rat sarcoma viral oncogene homolog *(KRAS)* and the Tumoral Protein p53 *(TP53)* genes. In particular, commercial competitive allele-specific TaqMan® PCR (castPCR™) technology, as well as TaqMan® and ZEN™ assays, have been evaluated for EGFR p.L858R, p.T790M, p.L861Q point mutations and in-frame deletions Del19. Specificity and sensitivity have been determined on cell lines DNA, plasmatic circulating DNA of lung cancer patients or Horizon Diagnostics Reference Standards. To show the multiplexing capabilities of this technology, several multiplex panels for EGFR (several three- and four-plexes) have been developed, offering new "ready-to-use" tests for lung cancer patients.

## Introduction

**Fig 1:**
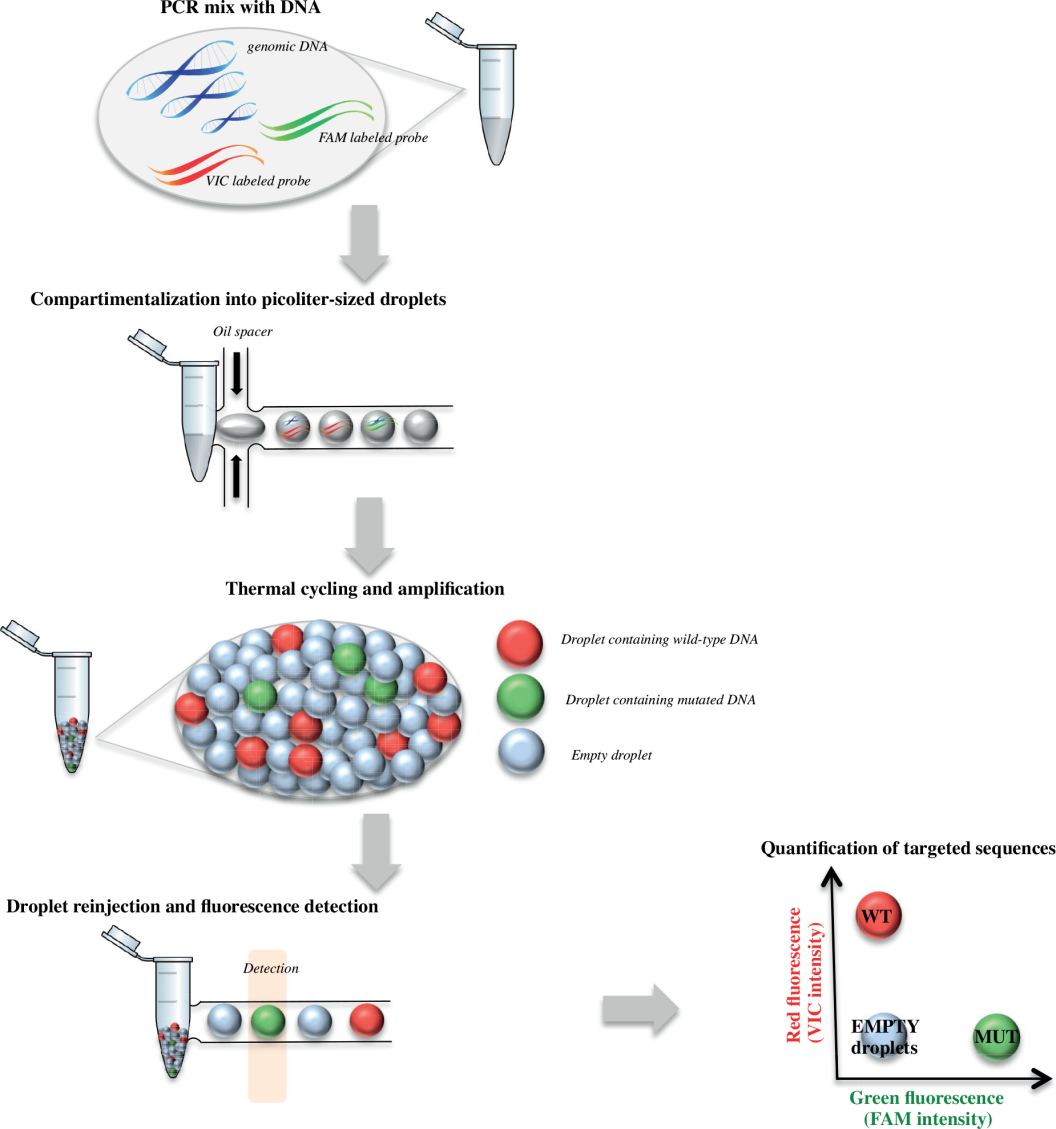
Experimental workflow for picoliter droplet-based digital PCR. An aqueous phase containing PCR reagents, probes, primers and genomic DNA (fragmented if using DNA extracted from cell culture or frozen tissues) is partitioned into droplets using the RainDrop® Source machine (RainDance Technologies, Billerica, US). After thermal-cycling, droplets are re-injected into RainDrop® Sense instrument, permitting the fluorescence detection of each individual droplet. Analysis is finally performed using the RainDance Technologies Analyst software. Empty droplets correspond to droplets containing no targeted DNA. *WT*, *droplets containing wild-type DNA; MUT*, *droplets containing mutant DNA*.

Droplet-based digital PCR (dPCR) represents an increasingly applied method for quantification and detection of nucleic acids [1]. This technique is based on the compartmentalization and amplification of single DNA molecules into up to millions of individual identical compartments (droplets here), so that each compartment contains either zero or one copy of the target DNA following a Poisson distribution [2, 3]. After allele-specific PCR reaction in presence of fluorogenic probes, the counting of positive and negative events reveals the number of copies of target DNA initially present in the tested sample (Fig 1). The sensitivity of dPCR is limited mainly by the number of droplets that can be analyzed and the false positive (FP) rate of the mutation detection assay [4]. Thanks to its higher sensitivity and accuracy in comparison to traditional PCR analysis methods, dPCR is increasingly applied in clinical research for diagnostic, prognostic and predictive evaluation of the disease [5–7].

One of the most attractive fields of dPCR application is cancer research [8, 9]. Due to its ability to quantify small amounts of mutated DNA molecules (MUT) among a large number of wild-type molecules (WT, non-mutated), this technique permits the detection of rare or low abundant alleles in cancer patient samples [10, 11]. In particular, dPCR allows the detection of mutations in circulating tumor DNA from liquid biopsy (e.g. blood plasma, serum, urine) [12, 13], enabling a non-invasive approach for accurate monitoring of disease progression and treatment efficacy [7].

Strategies based on dPCR generally share similar workflow consisting of, first, the identification of at least one target mutation in the patient tumor and, secondly, the specific detection of the identified mutation(s) to evidence the presence of circulating tumoral DNA (ctDNA) [14]. In this context, due to the high quantity of potential cancer specific mutations, there is a constant need for new and "ready-to-use" assays. The assays should allow to track mutated allele with high sensitivity and quantitativity permitting the follow-up of the evolution of a patient’s cancer. Many commercial quantitative PCR (qPCR) assays have been developed, and their optimization for dPCR could represent a strong benefit for cancer diagnostic and research.

**Fig 2:**
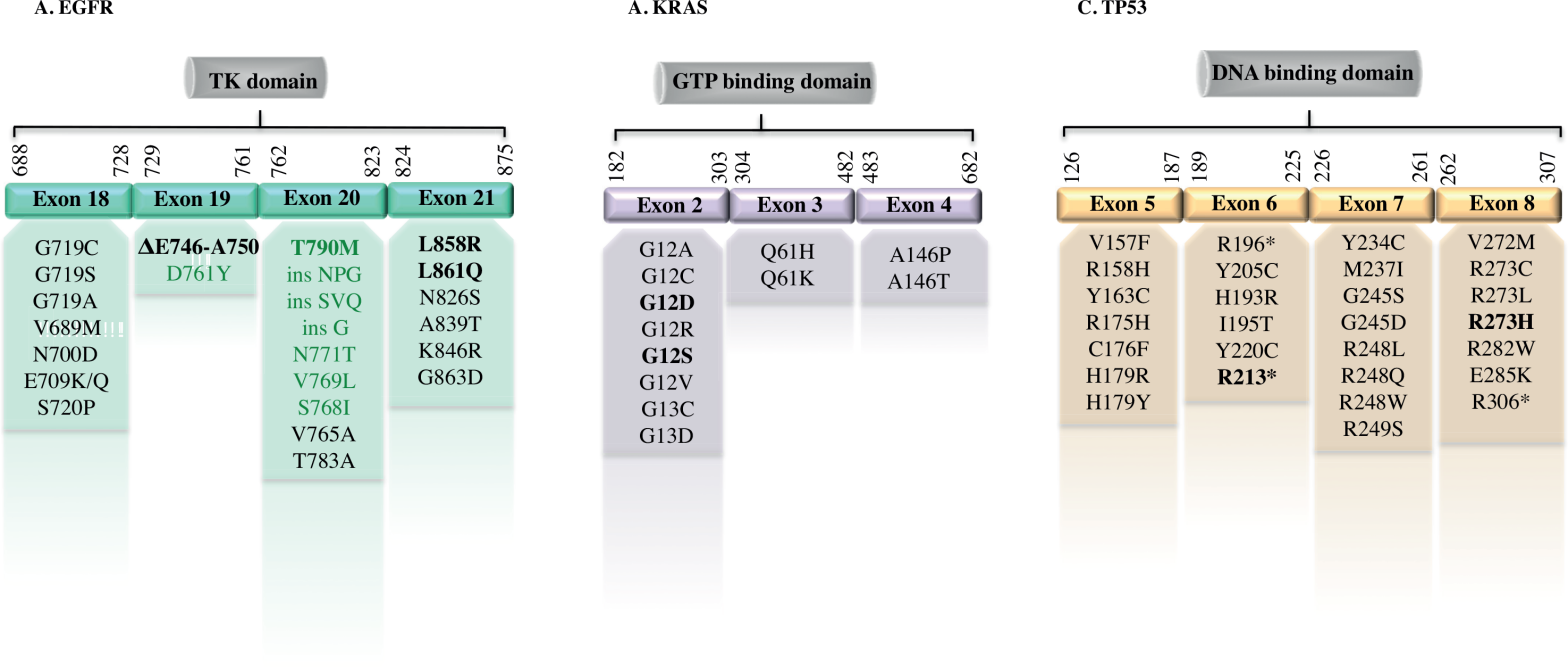
Mapping of most frequent *EGFR*, *KRAS* and *TP53* mutations. In *EGFR* gene (A), most of the mutations occur within Tyrosine Kinase (TK) domain (in light green, those associated with drug resistance). In *KRAS* (B), the most frequent mutations are located in exon 2 (corresponding to its GTP binding domain), while in *TP53* (C) they are mainly located in its DNA binding domain. In bold, mutations targeted in the study.

Lung cancer is the first leading cause of death worldwide (more than one million and half deaths in 2012 [15]). The most recurrent genetic alterations in non-small cell lung cancer (NSCLC, 85% of lung cancers) consist of mutations in the epidermal growth factor receptor (*EGFR*) gene, leading to uncontrolled cellular proliferation, inhibition of apoptosis and thus, tissue growth and cancer. The most common mutations of *EGFR* are located in exons 18, 19, 20, 21 of its Tyrosine Kinase (TK) domain (Fig 2A). Gefitinib (Iressa®) and Erlotinib (Tarceva®) are first-line selective inhibitors of EGFR TK domain (Tyrosine Kinase Inhibitors, TKI), and are effective in NSCLC population of patients [16, 17]. In this context, it has been established that some mutations in the *EGFR* gene are responsible for sensitivity or resistance to these treatments [18, 19]. The missense point mutation p.L858R (c.2573T>G) in exon 21 and the in-frame deletion in exon 19 account for almost 80% of all clinically important mutations related to TKI sensitivity [20]. Another point mutation in EGFR exon 21 present in 2% of NSCLCs is p.L861Q (c.2582T>A) [21]. Importantly, the “second-site” point mutation p.T790M (c.2369C>T) in EGFR exon 20 can emerge during treatment and confers drug resistance to tumor cells [6, 22].

Another worldwide common cancer is colorectal cancer, where 40% of colorectal adenocarcinoma are *KRAS* (v-Ki-ras2 Kirsten rat sarcoma viral oncogene homolog)-mutated (Fig 2B) [5, 23]. It has been demonstrated that only patients with WT *KRAS* tumors benefit from anti-EGFR monoclonal antibodies treatment in colorectal cancer, while *KRAS* mutations confer resistance to anti-EGFR therapy [5, 24, 25]. Moreover, *KRAS* mutated subclones have been highlighted in several adenocarcinomas by different studies [26, 27]. Hence, analysis of *KRAS* mutational status became crucial for therapy design [8, 28].

Finally, we focused on *TP53* (tumor protein p53), a frequently mutated gene in cancer, being altered in approximately 50% of human malignancies [29]. We characterized two assays for common mutations of this oncogene, p.R213* (c.637C>T) in exon 6 and p.R273H (c.818G>A) in exon 8 of *TP53* gene (Fig 2C) [30, 31].

Herein, we optimized and compared different assays (including commercial ones) for main mutations in *EGFR*, *KRAS* and *TP53* genes by a digital PCR approach. The standardization of procedures for mutations detection could serve as an appropriate tool for tumor mutational evolution analysis.

## Materials and Methods

### Control DNA and cell lines

*EGFR*-mutant lung adenocarcinoma H1975 (c.2369C>T, p.T790M and c.2573T>G, p.L858R) and H1650 (del E746-A750) cell lines, *KRAS*-mutant A427 (c.35G>A, p.G12D) and LS123 (c.34G>A, p.G12S) cell lines, *TP53*-mutant HT-29 (c.818G>A, p.R273H) cell line were purchased from ATCC (Manassas, VA 20110, US). Cell lines bearing *EGFR* mutations were genotyped to confirm the presence of targeted mutations using Sanger sequencing (data not shown).

*TP53*-mutant DNA isolated from the SW-648 cell line (p.R213*, c.637C>T) was purchased from Cell Lines Service (CLS) Company (Eppelheim, Germany).

For p.L861Q (c.2582T>A) EGFR assay, a paraffin-embedded tissue was obtained from a patient with metastatic lung cancer (George Pompidou Hospital, Paris, France) in accordance with Cancer Institute recommendations.

Horizon Diagnostics^TM^ cfDNA, Multiplex FFPE and Multiplex gDNA Reference Standards were used for multiplex developments.

For details about cell lines and reference standard, refer to S1 Fig.

### Human blood samples and plasma isolation

Blood samples were collected from a cohort of patients with lung cancers (stade IIIB and IV). A written informed consent was obtained for all patients included in the study. The protocols for the use of blood samples were approved by the Ethic Commitee (CPP Ile-de-France II, n° 2013-06-21 SC). Four mL of blood were collected in EDTA tubes. The blood was centrifuged at 2,000g at room temperature for 15 minutes. Plasma were stored at -20°C and centrifuged a second time at 2,000g at room temperature for 15 minutes in an Eppendorf 5430R centrifuge before DNA extraction.

Plasma samples in EDTA tubes from healthy donors have been purchased from Biological Speciality Corporation (Bristol, PA 19007, US). DNA samples coming from two females (one non-smoker) and two males (one non-smoker) donors have been extracted as described in the next section from 2 mL of plasma.

### DNA extraction and fragmentation

Cell line DNA (from around three millions of cells) was extracted using QIAamp® DNA Mini Kit, while DNA from FFPE samples (patient’s sample or Multiplex FFPE Reference Standards) was isolated with QIAamp® DNA FFPE Tissue Kit (QIAGEN, Les Ulis, France) according to the manufacturer’s instructions. DNA from cell lines and DNA from FFPE samples were eluted in 200 μL or 100 μL of elution buffer, respectively.

Cell-free DNA [12] (cfDNA) was extracted from plasma samples using QIAmp® Circulating Nucleic Acid Kit (QIAGEN, Les Ulis, France) according to the manufacturer’s instructions, and resuspended into 50 μL of elution buffer.

The DNA quantity has been measured by Qubit® 2.0 Fluorometer (Qubit® dsDNA BR Assay kit for DNA from cell lines and HS Assay kit for cfDNA—Life Technologies-Thermo Fisher Scientific, Saint Aubin, France) [32].

All DNA used in the study were kept at -20°C before use.

Genomic wild-type and mutated DNA were fragmented with S220 Focused-Ultrasonicator sonicator (Covaris, Woburn, MA) to mean sizes of 600–800 base-pairs (bp). DNA sizes have been verified by LabChip® GX/GXII Microfluidic system (Perkin-Elmer, Villebon-sur-Yvette, France) using DNA assay 5K reagent kit (Perkin-Elmer, Villebon-sur-Yvette, France).

### qPCR bulk assays

Quantitative PCR on DNA extracted from patient samples and control DNAs were run in a final volume of 10 μL in 384 wells plate using the reagents final concentrations presented in S2 Fig, including final 1X castPCR™ assay, 0.2 and 0.8 μM for TaqMan® probes and primers, respectively, and 0.2 and 0.4 μM for ZEN™ probes and primers, respectively. Runs were performed on an ABI Prism 7900 HT sequence detection system (Applied Biosystems, Foster City, CA) using the thermo cycling conditions shown in S5 Fig. Results were analyzed with the SDS 2.3 software.

### NGS analysis and protocol

Sequencing libraries were prepared from cfDNA using Ion AmpliSeq™ Colon and Lung Cancer Research Panel V2 (Life Technologies-Thermo Fisher Scientific), following the manufacturer’s recommendations. The multiplex barcoded libraries were generated with Ion AmpliSeq Library kit v2 (4480442) from six μL of plasmatic cell-free DNA as input. Libraries were normalized using the Ion Library Equalizer kit (4482298). The pooled barcoded libraries (max. 96) were processed on an Ion Chef™ System using an Ion PI Hi-Q Chef Kit (A27198) and sequenced on an Ion Proton™ System using and an Ion PI Chip Kit v3 (A26771). The FASTQs sequencing data were processed and aligned to the human genome (hg19) using the Ion-Torrent Suite V4.2.1. We then applied the Base Position Error Rate method that detects mutations in a tested sample using a binomial test to compare at each base position the A,T,C,G, insertion and deletions counts with those obtained in 29 control samples. Among all tested base position in one sample, mutations were called for the most statistically significant positions determined with logit and adjusted boxplot outlier detection methods (manuscript under review).

### Picoliter droplet based digital PCR: emulsion generation, thermal-cycling and droplets analysis

All PCR assay mixes were prepared as shown in S2 Fig in a pre-PCR room to limit risks of contamination. CastPCR™, TaqMan® (both from Life Technologies-Thermo Fisher Scientific) and ZEN™ (Integrated DNA Technologies, Louvain, Belgique) probes were tested. In classic TaqMan® assay, the probe bearing VIC-fluorophore (λ_ex_ 538 nm / λ_em_ 554 nm) was designed to be specific to the WT allele, while the probe bearing FAM-fluorophore (λ_ex_ 494 nm / λ_em_ 518 nm) was able to specifically hybridize to the mutated sequence (S3 Fig presents the details for each assay). ZEN™ technology uses hydrolysis of double-quenched fluorogenic probes, with one probe bearing TET-fluorophore (λ_ex_ 521 nm / λ_em_ 536 nm) specific for the WT allele, and one probe bearing FAM-fluorophore for the mutated sequence (similarly to TaqMan® assays). With castPCR™ technology, the specificity to the mutated locus (if present) is determined by the primer, while a blocker is impeding probe’s hybridization to WT allele; a classic TaqMan® probe distant-located from targeted mutation is used as a reference for quantification of total target DNA (refer to S4 Fig for the description of these mutation detection assays). It is to note that we modified the classic castPCR™ protocol that permits to use probes bearing only FAM-fluorophore in a two-wells bulk analysis where mutant allele is tested in a single well and the total target DNA in another well. The use of a VIC-fluorescent assay targeting both alleles in a non-mutated common region and a FAM-fluorophore for the mutated allele, permitted to analyze simultaneously WT and MUT targets in one assay. This improvement permitted to handle qPCR experiments in one-well analysis, as well as to implement dPCR multiplex tests by varying probes concentrations.

DNA was added to the mix in a separate room, after been fragmented with S220 Focused-Ultrasonicator sonicator in order to avoid DNA viscosity and thus contingent coalescence. For cell-lines or FFPE extracted DNA, 20 to 60-nanograms (ng) were used. PCR reactions were prepared to final 25 μL volume, containing from 300 to 800 DNA copies/μL for cell lines DNA (except for the 0.01% dilution where 50 μL emulsions have been used). For plasma DNA, three to six μL of eluted sample was used per reaction, independantly of the DNA amount.

The mix was compartmentalized into droplets with the droplet generator RainDrop® Source (RainDrop® Digital PCR System, RainDance Technologies, Billerica, US) for production of five picoliter (pL)-droplets partitioning DNA into approximately five or ten million droplets dependent upon starting sample volume (occupancy rate λ ≈ 0.001 and 0.004 when applying 20 or 60 ng of DNA, respectively). Emulsions were collected into eight-strip PCR tubes (Axigen, VWR, Fontenay sous bois, France). The samples were thermal-cycled (refer to S5 Fig for thermocycling programs) using a BioRad® thermal cycler (MJ-Mini, S1000, or C1000 touch). Finally, samples were sealed with opaque flat caps (RainDance Technologies, Billerica, US) and transfered to the RainDrop® Sense instrument (RainDance Technologies, Billerica, US). Lasers within the RainDrop system are used to excite and read the FAM and VIC/TET fluorescence intensity of droplets. Data were then analyzed using the RainDrop Analyst data analysis software. For all dPCR experiments, droplets events have been normalized to five or ten milions droplets (the partition number theoretically expected), and the number of mutated-DNA containing droplets has been re-calculated after substraction of the Limit of Blank (LOB), typical of each probe.

For description of multiplex mutation detection assays, refer to S6 Fig.

### Statistical analysis

In conditions of limiting dilution, the distribution of DNA molecules has been shown to follow a Poisson statistics [3]. As a consequence, all events including FP counts follow this statistics, supposing that the number of FP should not change if using greater amount of input DNA as previously described [8]. The LOB and the Limit of Detection (LOD) of our assays could thus be determined using a Poisson statistical analysis as described by Milbury *et al* [33]. Statistical analysis were performed on Excel and Prism software (GraphPad Software, Inc.). Mutation titration series have been analyzed on MatLab. All data are presented in order to follow digital PCR MIQE guidelines [1].

## Results and Discussion

### Verification of linearity and sensitivity of the assays

Digital PCR has emerged as a highly effective technique for evaluation of tumor patients’ status. Thanks to its sensitivity, this method permits detection and quantification of rare sequences from low amount of starting material. Notably, droplet-based digital PCR allows the detection of mutations in circulating tumor DNA from liquid biopsies [34]. Nevertheless, the detection of different mutations demands the development of various assays, that should allow specific detection and quantification of each mutation. To avoid high costs and efforts associated with such developments, "ready-to-use" assays should be of high interest. Besides, these tests could as well broaden dPCR handling to non-expert users.

We thus assessed the utility and relevance of castPCR™ technology, recently developed by Life-Technologies-Thermo Fisher Scientific Company, compared to conventionaly used TaqMan® system for various *EGFR*, *KRAS* and *TP53* mutations [28]. We first tested them on fragmented DNA extracted from cell line harboring the corresponding mutations. In parallel, the assay has been run on fragmented WT genomic DNA for determination of the number of FP droplets (if present) of each assay.

**Fig 3:**
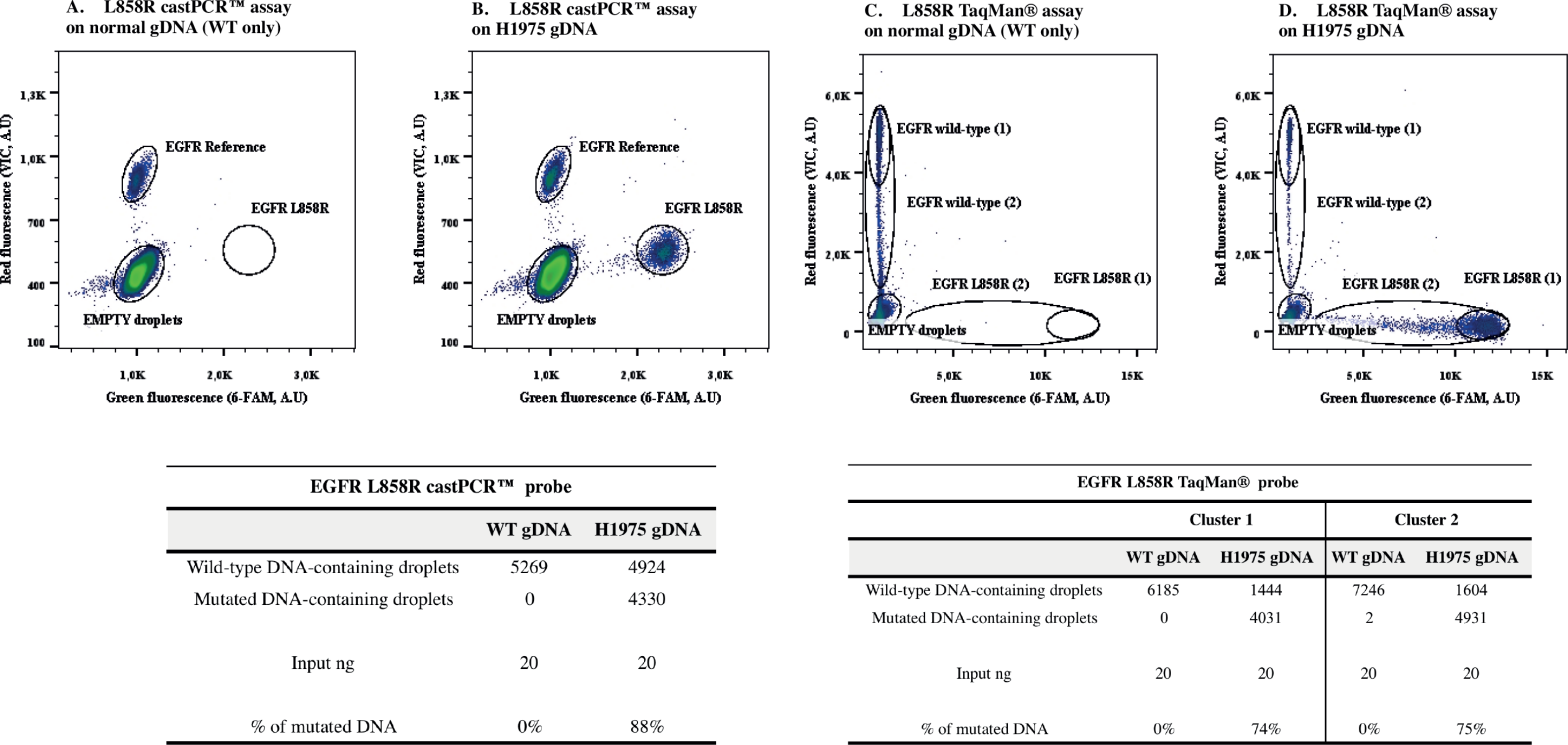
Examples of EGFR L858R castPCR™ and TaqMan® asssays. Plots obtained from dPCR analysis using EGFR L858R castPCR™ assay (panels A and B) and TaqMan® assay (panels C and D). As negative and positive controls, fragmented human wild-type genomic DNA (A and C) and H1975 cell line genomic DNA (B and D) have been used, respectively (see S6 Fig for probes/primers concentrations). In the lower tables, droplets counts from these experiments are listed. Input ng represents the amount of DNA used in dPCR, previously estimated by Qubit® 2.0 Fluorometer. *A*.*U*, *arbitrary units; WT*, *wild-type; Reference*, *wild-type + mutant DNA; gDNA*, *genomic DNA*.

An example of analysis obtained using EGFR p.L858R castPCR™ and TaqMan® is shown in Fig 3 (left and right panel respectively). In the tables present under the figures, the molecule count is listed. Input DNA was theoretically 20 ng for five millions of droplets. The amount of genomes (WT-containing droplets in WT DNA sample) obtained was similar for both assays (between 5000 and 6000 genomes). When looking to the results in mutated H1975 cell line DNA, we obtained almost the same amount of MUT-containing droplets (around 4000 molecules) with both assays. The number of WT+MUT -called “Reference” for the castPCR™ test- and WT containing droplet counts were also in agreement: 5000 molecules for (WT+MUT)-containing droplets with the castPCR™ test and 1000 for WT-containing droplets with the TaqMan® assay. The difference in the number of counts for the red-bearing fluorescent droplets is justified by the different technical operating principle of these two detection systems. Indeed, within the classic TaqMan® assay fluorescent probes are specific for the WT or the MUT sequence (both placed in exon 21 around p.L858R mutation): we obtained 4000 mutated molecules and 1000 WT-DNA containing droplets. Within castPCR™ system, a first set of primers and probes is directed specifically to the targeted mutation (with primers specific for p.L858R point mutation and a blocker of WT sequence, obtaining 4000 mutated molecules) and a second one targets a remote region (in exon 17) common to both WT and MUT sequences (thus detecting 5000 molecules, which is exactly the total amount of WT- and MUT-DNA containing droplets within the TaqMan® assay).

Furthermore, we noted that, for this particular assay, when using the L858R TaqMan® assay the cluster obtained was more stretched than the one obtained with castPCR™ probes (Fig 3C and 3D). In order to verify the pertinence of droplets counting, we designed a small (wild-type or L858R “1”) and a bigger cluster (wild-type or L858R “2”) and compared the number of genomes obtained with both designs. Since the counts and the percentage of mutation obtained were nearly similar, we concluded that we could take into account the small (“1”) cluster only.

With these assumptions, for the p.L858R detection, we evaluated castPCR™ test as more specific than TaqMan® systems, both for its specificity (determined by the WT-blocker and by the implementation of a reference assay far-away located from the analyzed mutation) and for clusters shape (which resulted easier to define compared to the stretched clustering of TaqMan® assay). Yet, castPCR™ test performance depends on targeted mutation/gene (i.e. both for KRAS p.G12S and TP53 p.R273H mutations the TaqMan® assay shown higher efficiency then castPCR™ test).

For the p.T790M resistance mutation of EGFR, we compared the castPCR™ with the ZEN™ assay (see Milbury *et al*. [33]), consisting of a LNA PrimerTime® dual-labeled DNA probe with two quenchers (S4 Fig). As shown by mutation titration series presented later in the text, ZEN™ assay demonstrated higher sensitivity (up to 0.01%) than the castPCR™ assay.

In S7 Fig, the comparison between castPCR™ and TaqMan® assays is shown for p.G12S mutation of KRAS, and p.R273H mutation of TP53.

**Fig 4:**
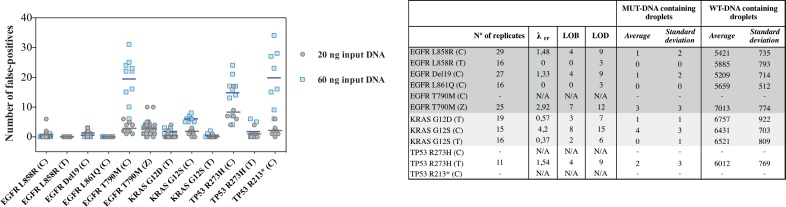
False-positive evaluation in negative controls (human wild-type genomic DNA). In order to assess the false-positive (FP) events detected in negative control samples, we analysed by dPCR a collection of human wild-type only samples (genomic DNA, refer to S1 Fig for details) with the EGFR, KRAS and TP53 assays described. We used two different amounts of DNA input (20 and 60 ng, depicted by circles and squares respectively). The scatter plot displays the low dynamic range detection of three castPCR™ probes (EGFR p.T790M, TP53 p.R273H and p.R213*), where the number of FP events increased when using 60 ng of starting DNA material (lines represent the mean for each assay). At right, the table shows the LOB and LOD estimation of all assays (refer to [33] for precise *formula*), calculated from the λ_FP_ of each test (where λ_FP_ is given by the mean number of false-positives obtained in all experiments realized with 20 ng input DNA). Mean value and standard deviation for each FP measurement for the different assays are shown, both for WT and MUT-DNA containing droplets. *(C)*, *castPCR™ probes; (T)*, *TaqMan® probes; (Z)*, *ZEN™ probes; N°*, *number; FP*, *false-positive; LOB*, *Limit of Blank; LOD*, *Limit of Detection; N/A*, *not applicable*.

Five other most frequent mutations of KRAS (p.G13D, p.G12V, p.G12R, p.G12A, p.G12C) were tested with both castPCR™ and classic TaqMan®. Some castPCR™ assays targeting less frequent mutations (p.Q61Q, p.Q61H, p.A146T for KRAS, p.G469A for BRAF and p.Q61R for NRAS, not available with TaqMan® system) were also validated by dPCR in a context of clinical follow-up (data not shown). Furthermore, we tested castPCR™ technology for three other common mutations of TP53 (p. R175H, p.R248Q, p.R273C) and obtained results comparable to the one described for p.R273H and p.R213* (data not shown). From these testings, we could conclude that classic TaqMan® technology rather than castPCR™ was a better option for the described dPCR assays, due to lower FP rate (refer to Fig 4).

Subsequently, to evaluate the sensitivity of each assay, we determined the mean FP droplet event frequency from a series of wild-type-only control samples (where the FP corresponds to the number of droplets that fall into the MUT-containing droplet cluster in a non mutated sample, which *a priori* should not present those events) (refer to supplementary data from [8] and [33]).

For these determination, we performed multiple experiments on a set of WT genomic DNA samples, ranging from 20 to 60 ng of starting DNA material, supposing that the number of FP should not change if using greater amount of input DNA (as previously described [8]).

For most assays, FP rate did not varied relative to the quantity of input DNA, except for castPCR™ assays targeting EGFR p.T790M, TP53 p.R273H and p.R213* where we observed a higher FP rate if using 60 ng-input (Fig 4). A possible explanation, at least for T790M castPCR™ assay, is the difficulty of primer design around this point mutation. Though, it is known that the region of interest is GC-rich. Moreover, it contains a single nucleotide polymorphism eight nucleotides upstream the mutation site and a repetitive sequence element close to it. However, for the p.T790M assay using ZEN™ technologies, previously described by Milbury *et al*. [33], the number of FP was independent of DNA amount (see Fig 4).

After this observation, for the assay where the FP did not varied in function of the quantity of input DNA, a Poisson model was fit to the data with a parameter, **λ**, which is the mean of the Poisson distribution, and evaluating the 95% one-tailed upper limit of the model distribution.

From λ_FP_, we calculated LOB and LOD for all the assays (for further information on statistical analysis, refer to [33]). These metrics were useful for description of sensitivity of our tests (right panel in Fig 4). In particular, LOB was helpful for evaluation of mutation percentage, as samples were considered positive when the number of observed droplets was higher than LOB value for the considered test. Yet, this was possible only when FP rate was not depending from input DNA amount (LOB calculation was not possible–N/A in table of Fig 4– for castPCR™ assays targeting EGFR p.T790M, TP53 p.R273H and p.R213*).

**Fig 5:**
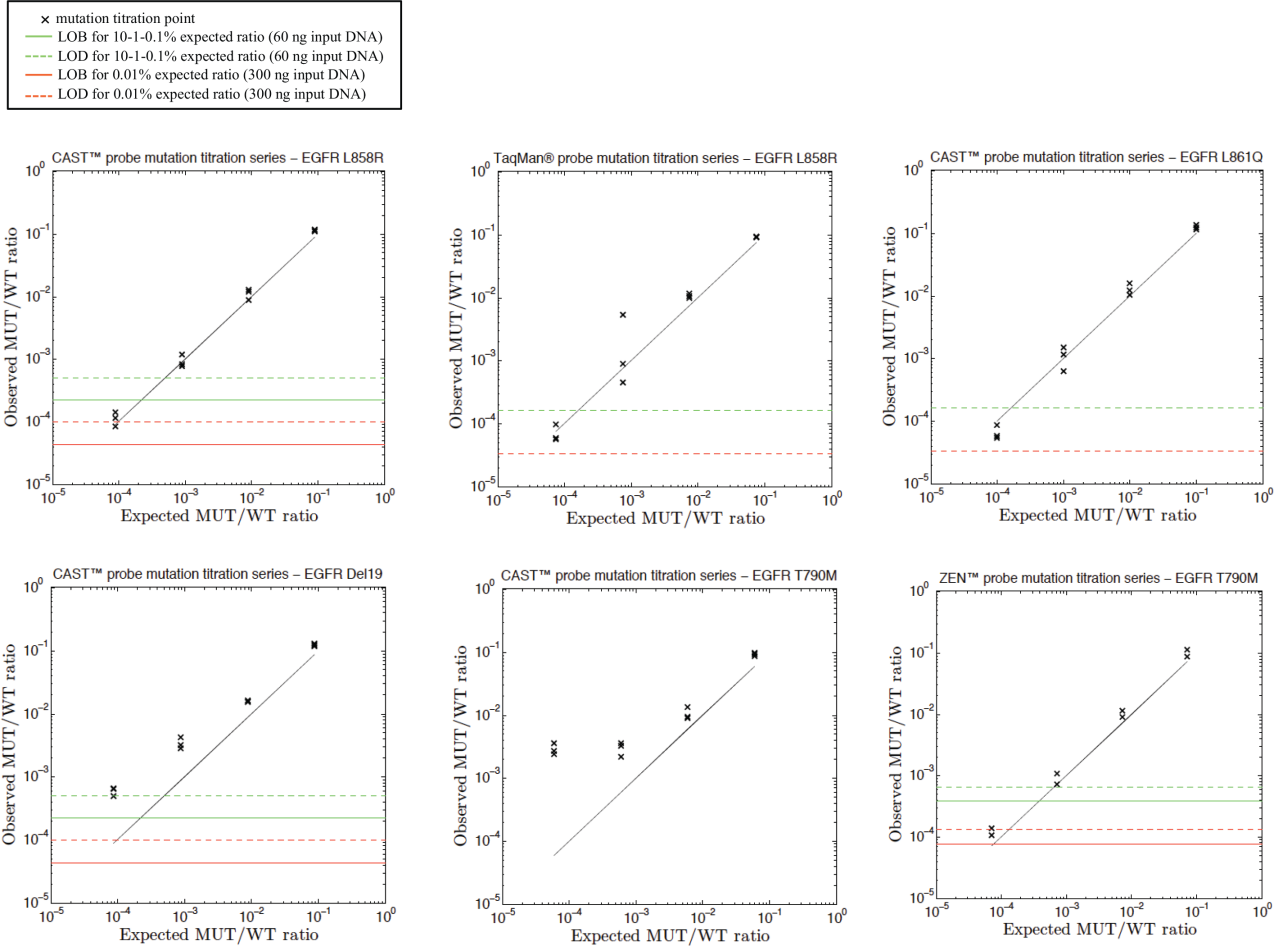
Examples of titration series with EGFR castPCR™, ZEN™ and TaqMan® probes. Serial dilutions of L858R, T790M, Del19, or L861Q mutated DNA (extracted from H1975 cell line, H1650 cell line or FFPE tissue, respectively) in human wild-type genomic DNA. Individual data points are displayed for independent replicates. The expected mutant to wild-type ratio (black line) is shown. Green continuous and dashed lines represent LOB and LOD values, respectively, evaluated from droplets falling into the mutated-DNA cluster and analyzed in a WT gDNA sample for each replicate. For the lowest titration point (0.01%), we used a higher amount of input DNA. Thus, corresponding LOB and LOD values are represented by red lines. Since number of FP was increasing with quantity of input DNA for EGFR p.T790M castPCR™ test, LOB and LOD calculation could not be performed (refer to [8]).

**Fig 6:**
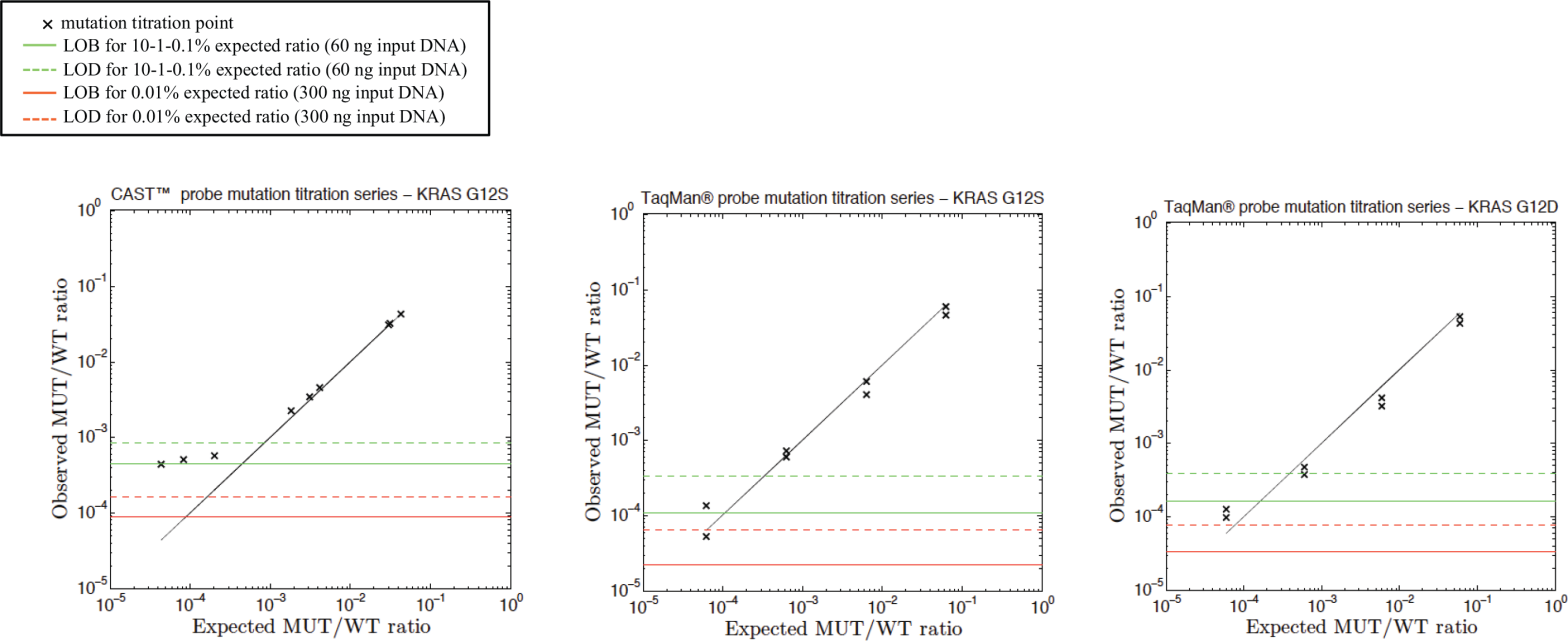
Examples of titration series with KRAS castPCR™ and TaqMan® probes. Serial dilutions of G12D and G12S mutated DNA (extracted from A427 and LS123 cell lines, respectively) in human wild-type genomic DNA. Individual data points are displayed for independent replicates. The expected mutant to wild-type ratio (black line) is shown. Green continuous and dashed lines represent LOB and LOD values, respectively, evaluated from droplets falling into the mutated-DNA cluster and analyzed in a WT gDNA sample for each replicate. For the lowest titration point (0.01%), we used a higher amount of input DNA. Thus, corresponding LOB and LOD values are represented by red lines.

**Fig 7:**
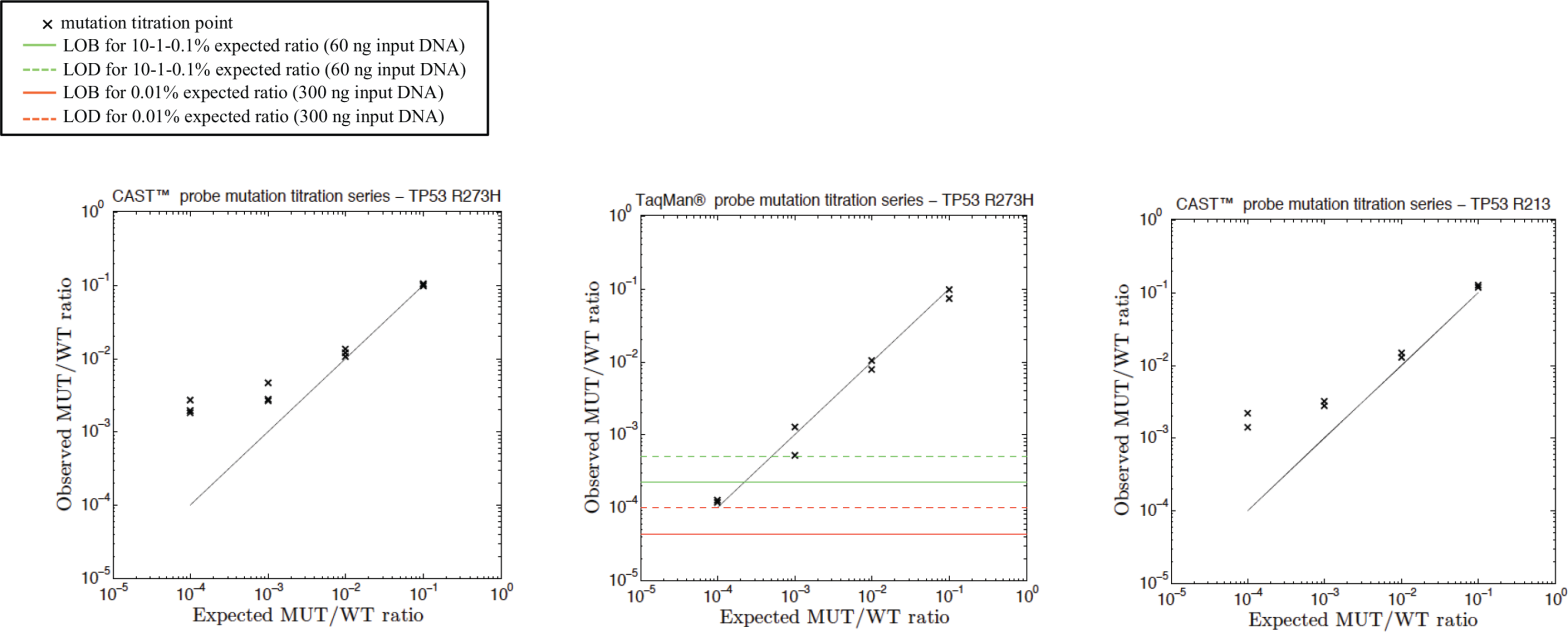
Examples of titration series with TP53 castPCR™ and TaqMan® probes. Serial dilutions of TP53 p.R273H and p.R213* mutated DNA (extracted from HT-29 and SW-684 cell lines, respectively) in human wild-type genomic DNA. Individual data points are displayed for independent replicates. The expected mutant to wild-type ratio (black line) is shown. Green continuous and dashed lines represent LOB and LOD values, respectively, evaluated from droplets falling into the mutated-DNA cluster and analyzed in a WT gDNA sample for each replicate. For the lowest titration point (0.01%), we used a higher amount of input DNA. Thus, corresponding LOB and LOD values are represented by red lines. Since number of FP was increasing with quantity of input DNA for TP53 p.R273H and p.R213*castPCR™ test, LOB and LOD calculation could not be performed (refer to [8]).

A mutation titration series was then performed in duplicate or triplicate for evaluation of linearity and sensitivity of assays. For each test, we diluted DNA from corresponding mutated cell line in WT DNA, up to 0.01%. Higher dilutions were not tested further to be coherent with the amount of input DNA expected from clinical samples. Data are shown in Fig 5 for EGFR assays, in Fig 6 for KRAS, and in Fig 7 for TP53 assays. All assays are globally sensitive up to the last titration sample (0.01%), except for the castPCR™ tests targeting EGFR p.T790M, TP53 p.R273H and TP53 p.R213*, which have a sensitivity limited at 0.1%. Despite numerous experiments we performed in order to improve those tests, we could not gain in sensitivity.

The mean droplet counts from independent experiments for each mutation titration test are listed in S8 Fig.

### Test of EGFR assays on patient samples and implementation of multiplex formats

**Fig 8:**
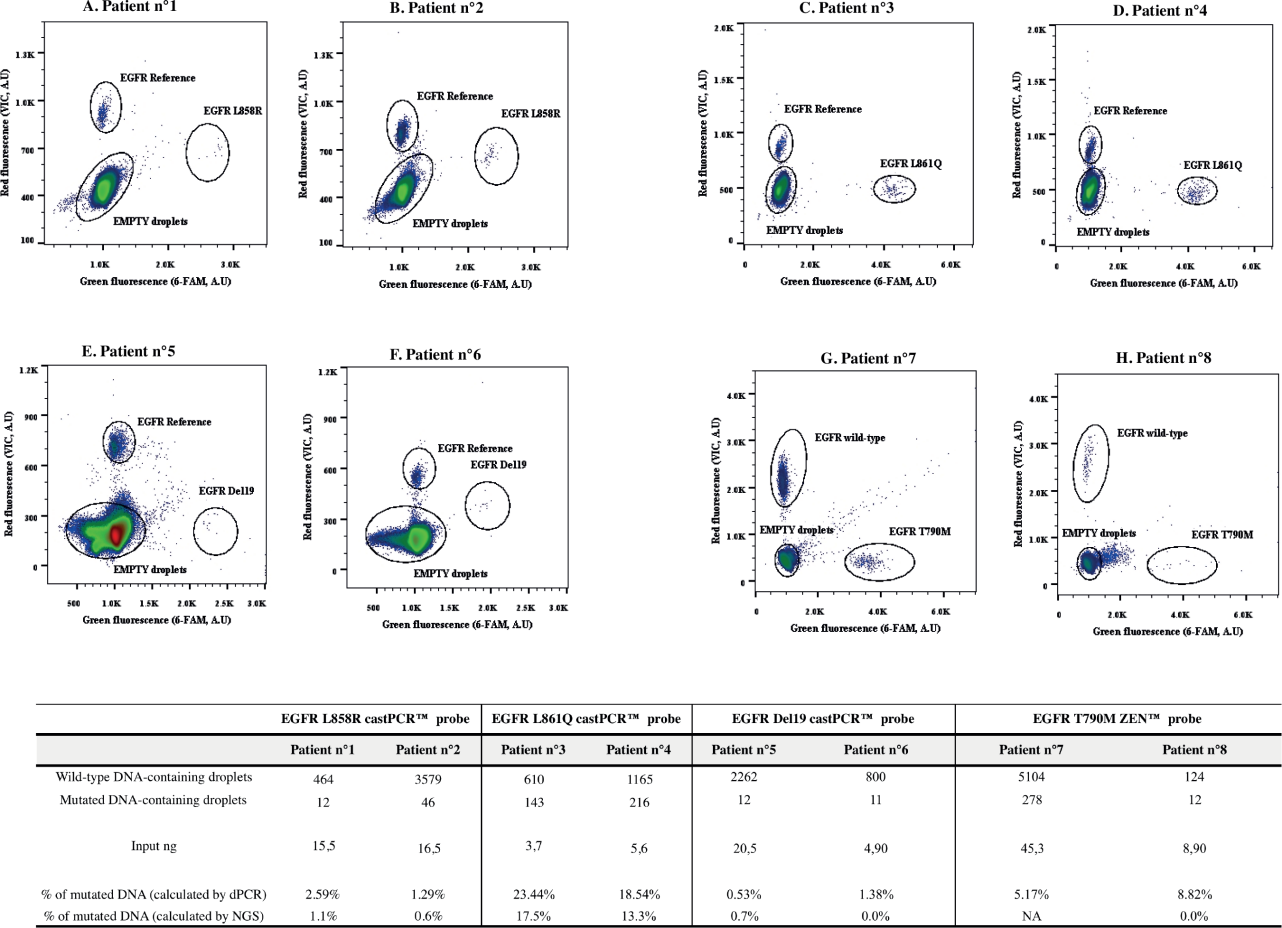
EGFR L858R, L861Q, Del19 and T790M mutation screening on lung cancer patients plasma using dPCR two-plex assay. Two-plots analysis on plasma DNA samples, whose initial tumor specific mutation has been previously determined by NGS on the tumor tissue. In the table, event counts from each experiment are listed. Input ng represents the amount of DNA used in dPCR, previously estimated by Qubit® 2.0 Fluorometer (three μL were used for each sample). Measured allelic frequencies are given for dPCR and NGS analysis. *Reference*, *wild-type + mutant DNA; NA*, *not analyzed; A*.*U*, *arbitrary units*.

Following characterization of EGFR castPCR™ tests, we evaluated their applicability on eight plasma DNA of lung cancer patients (stade III and IV). Mutation present in the tumor has been determined by Next Generation Sequencing (NGS) at the Georges Pompidou Hospital (Ion-Torrent, Thermo-Fischer, and AmpliSeq Colon and Lung cancer panel v2). Two plasmatic DNA samples have been tested for each mutation with the duplex panel (Fig 8). Three to six μL of DNA, whose amount has been previously estimated by Qubit® HS kit (from 3.7 to 45.3 ng, as shown in the lower table of Fig 8), were used as input for the test. Different percentages of mutated alleles have been obtained for each patient (from 0.53% to 23.44%). It has to be noted that the position of Del19 cluster could shift, probably depending on the number of nucleotides deleted in each sample (Deletions19 assay is a pool of nineteen different deletions on the *EGFR* exon 19).

The castPCR™ assays were run in a bulk qPCR experiment using the same DNA samples. As shown in S11 Fig, over the eight samples detected positive by dPCR, only four samples could be detected by qPCR, reflecting the fact that dPCR represents a powerful tool for detection of low mutation load in tumor samples.

**Fig 9:**
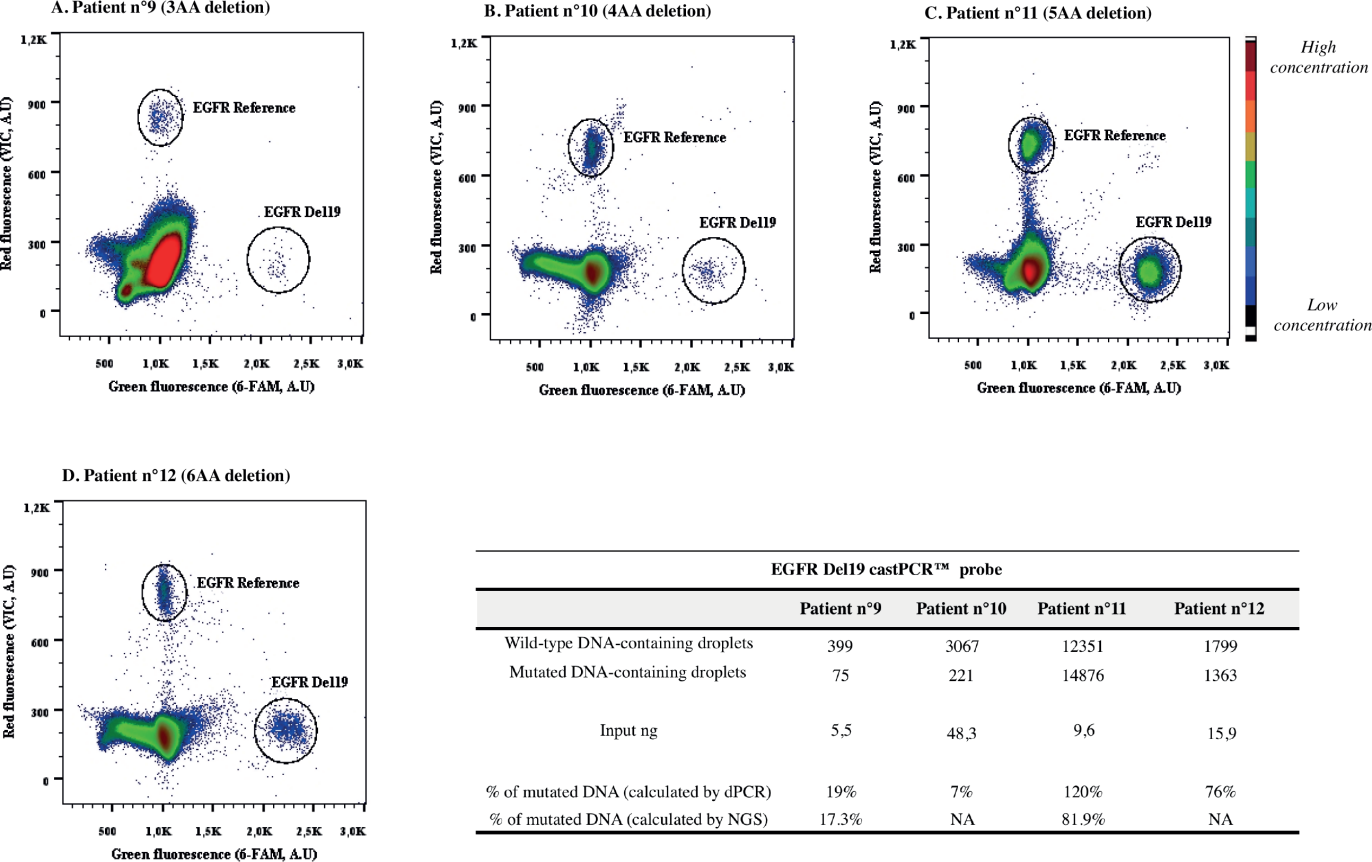
EGFR Del19 screening on lung cancer patients plasma using two-plex assay. These four plots were obtained from dPCR analysis on DNA extracted from plasma of lung cancer patients. The use of Del19 castPCR™ probe permitted to screen samples containing different deletions on exon 19 (of three, four, five and six amino acids, in panel A, B, C, D, respectively). In the table, event counts from the single experiments are listed. Input ng represents the amount of DNA used in dPCR, previously estimated by Qubit® 2.0 Fluorometer (three μL were used for each sample). Measured allelic frequencies are given for dPCR and NGS analysis. *Reference*, *wild-type + mutant DNA; NA*, *not analyzed; A*.*U*, *arbitrary units; AA*, *aminoacids*.

Besides, we tested EGFR Del19 assay on samples harboring different deletions for veryfing the specific targeting of this assay. We tested circulating DNA extracted from four plasma of lung cancer patients, which presented deletions of three, four, five or six amino acids. Three μL of DNA were used as input for the test (from 6 to 48 ng total). Two-plex plots of digital PCR experiments are shown in Fig 9. Percentage of mutated DNA was different in all samples, depending on tumor heterogeneity, on treatment line and time between blood sampling and start of treatment (plasma from patient 1 and 2 have been sampled at the beginning of the treatment; for patient 3, eight weeks after beginning of the treatment, while for patient 4, three weeks after beginning of the treatment) [35–37].

In this context, castPCR™ L858R, L861Q, Del19 and ZEN™ T790M assays have been successfully tested on ctDNA from plasma of lung cancer patients in a clinical study that will be submitted soon.

**Fig 10:**
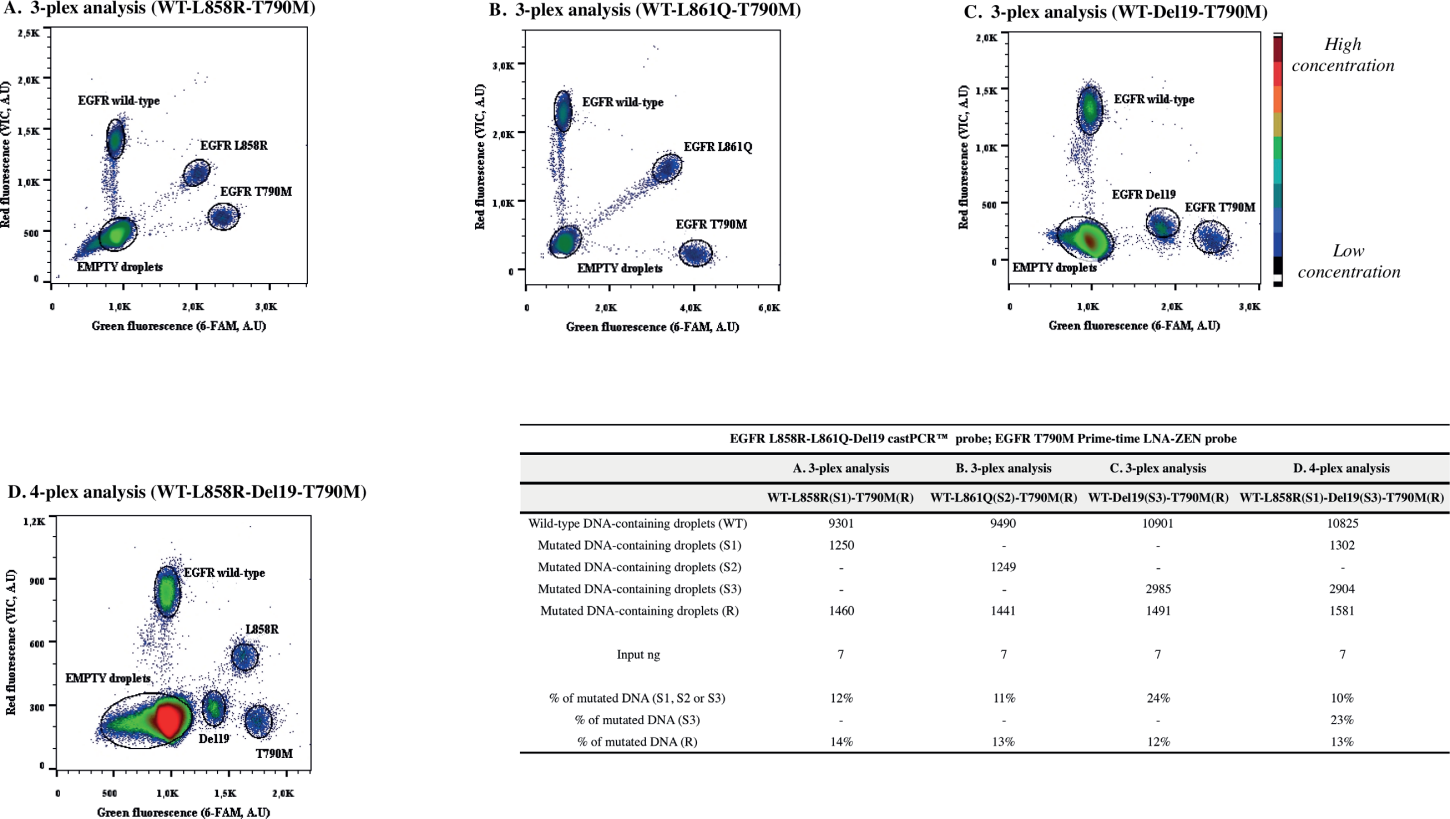
Multiplex assays for the most frequent *EGFR* mutations. In panel A, B and C, 2D-plots of the three-plex for follow up of the three sensitivity mutations with the T790M resistance mutation. The four-plex is shown in panel D. A pool of fragmented DNA extracted from two cell lines (H1975 harboring L858R and T790M mutations, H1650 harboring Del19 mutation), DNA from FFPE sample (for L861Q mutation) and fragmented wild-type only genomic DNA was used as input. A mix of mutation-specific VIC and/or 6-carboxyfluorescein cast™ and ZEN™ probes was optimized. In the table, event counts from the single experiments are listed (input ng represents the amount of DNA used in dPCR, previously estimated by Qubit® 2.0 Fluorometer). *A*.*U*, *arbitrary units; WT*, *wild-type; S*, *sensitivity mutation; R*, *resistance mutation*.

Furthermore, Fig 10 describes multiplex assays for the most recurrent *EGFR* mutations (for final concentrations and vendors of reagents used for multiplex test development, refer to S2 Fig for mix components and to S6 Fig for probes/primers concentrations–“four-plex assay panel”). Panel A, B and C show three-plex assays targeting the wild-type sequence, either one of the three sensitivity mutations (p.L858R, p.L861Q, Del19) as well as the resistance mutation p.T790M. Panel D presents a four-plex assay targeting the wild-type sequence, the two most recurrent sensitivity mutations of EGFR (p.L858R and Del19, accounting for around 80% of all mutations) and the p.T790M resistance alteration. A mixture of distinct probes at various concentrations permitted to isolate clusters corresponding to the presence of each mutation [8, 38] (refer to S6 Fig). A pool of DNA consisting of DNA extracted from the two cell lines H1975 and H1650, FFPE sample and genomic WT-only DNA was used as input for multiplex assays. As control, we performed the four-plex analysis on a pool of DNA, taking off the DNA from H1975 (S9A Fig) or DNA from H1650 (S9B Fig). Since the false positive occurrence and background signal differ in those panels from the corresponding duplex analysis, we calculated the LOB and LOD values for all multiplex test (S10 Fig).

Moreover, DNA from healthy subjects has been analyzed with the developed EGFR assay panels. While no MUT-containing DNA has been detected when using the duplex panel (except for one sample–M-NS-01 in L858R panel–; still its mutated allelic fraction was lower than LOD value), we noticed some background for three and four-plex tests (S11 Fig).

DNA samples previously analyzed using the two-plex assays (refer to Fig 8) were also tested using the multiplex assays. The allelic fractions calculated from this new analysis were comparable to the one observed by two-plex analysis. The fact that we observed a small discrepancy between the mutation load obtained when using the different panels can be justified by the higher background signal of three/four-plex when compared to the two-plex tests. In the case of the Del19 multiplex, it has to be underlined that a better quality in terms of clusters separation could potentially have been obtained using a Del19 castPCR™ assay bearing a VIC fluorophore (not commercially available at the time of manuscript writing). Moreover, since the assay is ready-to-use, we could not change primers and probes concentrations separately for improve clusters spatial resolution.

Even if optimization could be performed on these assays, such multiplex panels would be pertinent for the simultaneous detection of different alterations, meanwhile consuming a minimum quantity of patient sample [8]. Moreover, they might allow the detection of resistance mutation T790M while measuring the sensitivity one at the same time (S11 Fig). Furthermore, these multiplexed assays can not be tested by classic bulk experiments in just one assay.

### Confirmation of multiplex assays on commercially available DNA

**Fig 11:**
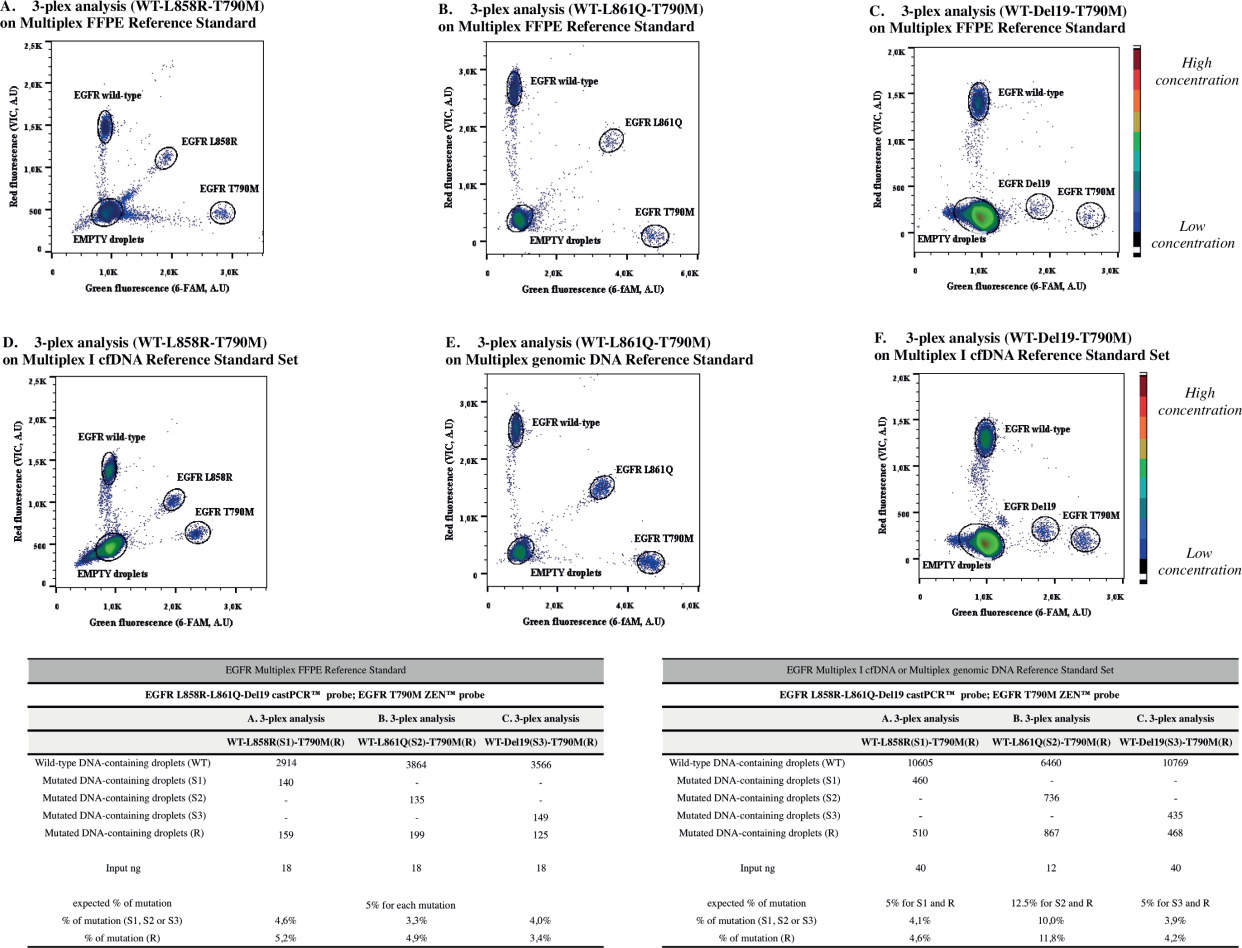
Multiplex panel for the most common *EGFR* mutations using HdX™ Reference Standards. A three-plex panel for follow-up of the 3 EGFR sensitivity mutations with the T790M resistance mutation. As input, DNA from FFPE Reference Standards (R.S.) (panels A, B, C), Multiplex I cfDNA (panels D and F) and Multiplex genomic DNA (panel E) from Horizon Diagnostics. The FFPE R.S. contains 50% of genomic DNA and 5% of each mutation, while while the Multiplex DNA is engineered from mutant cell lines for generation of 12.5% *EGFR* allelic frequency for the four mutations. Multiplex I cfDNA provides a set containing fragmented DNA in a range of low allelic frequencies (from 0.1% to 5%): we showed here only the 5% allelic frequency DNA. In the table, event counts from the single experiments are listed (input ng represents the amount of DNA used in dPCR, previously estimated by Qubit® 2.0 Fluorometer). *A*.*U*, *arbitrary units; WT*, *wild-type; gDNA*, *genomic DNA*.

In the interest of further confirming the pertinence of the developped multiplex assays, we ran the three-plex assays containing p.L858R or p.L861Q or Del19 and p.T790M mutations on three different EGFR Horizon Diagnostics (HDx™) Reference Standards (S1 Fig). The HD850 EGFR Gene-Specific Multiplex FFPE Reference Standard (with 5% Allelic Frequency) has been manufactured from five engineered *EGFR* mutant cell lines, mixed and formalin fixed-paraffin embedded, in order to generate a precise allelic frequency multiplex sample (5% of p.L861Q, ΔE746 –A750, p.L858R, p.T790M, p.G719S) (Fig 11A, 11B and 11C). The HD780 Multiplex I cfDNA Reference Standard Set was manufactured from engineered human cancer cell lines, and subsequently fragmented to an average size of 160bp to resemble cfDNA from human plasma (Fig 11D and 11F). The HD802 EGFR Gene-Specific Multiplex gDNA Reference Standard has been developed from four engineered *EGFR* mutant cell lines, generating a multiplex sample containing 50% of genomic DNA and 12.5% of p.L861Q (Fig 11E), 12.5% of ΔE746 –A750, 12.5% of p.L858R and 12.5% of p.T790M.

As shown in Fig 10, the obtained percentage of mutated alleles were close to those expected from HdX™ Reference Standard, confirming the feasibility and applicability of the characterized assay.

In conclusion, with this work we report the performance of the developed multiplex assays for various *EGFR*, *KRAS* and *TP53* mutations using the picoliter droplet-based dPCR system. Following the emergent need of rapid and "ready-to-use" tests for diagnostic, our goal has been to characterize three types of technologies, the castPCR™, TaqMan® and ZEN™probes as well as to develop pertinent EGFR multiplex assays. The results described revealed a good sensitivity for several castPCR™ and all TaqMan® probes. With some probes, we could detect one mutated DNA-containing droplet into 10.000 droplets (0.01% of sensitivity), while other tests (EGFR T790M, TP53 R273H and R213* castPCR™ assays) showed sensitivity untill 0.1%. Our results also show that these droplet based digital PCR represent a pertinent tool for circulating tumor DNA patients’follow-up and rare mutations detection in cancer research. Finally, this study presents the first multiplex (three-plex and four-plex) panel for the most common sensitivity mutation (p.L861Q, p.L858R and Del19) and the resistance mutation p.T790M. Further validations of these assays should now be performed in a context of a large cancer research study.

## Supporting Information

S1 Fig Cell lines details. For p.L861Q EGFR assay, DNA from tumor collection available in the laboratory has been used (refer to Methods part). For p.R213* (637C>T) TP53 mutation, DNA was directly purchased from Cell Lines Service (CLS) Company. Horizon Diagnostics^TM^ cfDNA, Multiplex FFPE and Multiplex gDNA Reference Standards have also been tested.
(PDF)

S2 Fig dPCR reagent components. (PDF)

S3 Fig EGFR, KRAS and TP53 assays details. Main information about castPCR™, TaqMan® and ZEN™ assays are reported in the tables. The transcripts we refered to are NM_005228.3 for *EGFR*, NM_033360.2 for *KRAS* and NM_000546.4 for *TP53* gene. *“MGBNFQ” refers to the minor groove binder non-fluorescent quencher; ** Proprietary information of Life Technologies-Thermo Fisher Scientific (sequences of probes and primers are not furnished).
(PDF)

S4 Fig Competitive Allele-Specific Taqman® PCR (castPCR™), Taqman® Mutation Detection Assays and ZEN™ Internal Quencher system. CastPCR™ technology (upper panel) permits specific amplification of one allele type (bringing the mutation) while an MGB blocker suppresses the wild-type sequence (if present) at the mutation site. As reference, a TaqMan® system permits the amplification of wild-type DNA further away from the targeted mutation (e.g. about 20.000 nucleotides for EGFR probes) (refer to http://www.appliedbiosystems.com/absite/us/en/home/applications-technologies/real-time-pcr/castpcr.printable.html for more information). The TaqMan® technology (lower left figure) permits the detection of the mutation by specific match of fluorescent probe with the sequence in which the mutation resides. ZEN™ Internal Quencher (lower rigth figure) system represents an original modification developed by IDT which helps in lowering background and increasing signal than traditional methods (refer to http://eu.idtdna.com/pages/products/gene-expression/custom-qpcr-probes for further information).
(PDF)

S5 Fig PCR Cycling Conditions. * For KRAS TaqMan® probes, an annealing temperature of 64°C has been used.
(PDF)

S6 Fig Multiplex mutation detection assay description. The amount of probes and primers used for duplex, triplex and quadruplex assays for the three targeted genes is shown. Since castPCR™ assays are selt as read-to-use by Life Technologies-Thermo Fisher Scientific company, we could not specifiy the final concentration (expressed in μM).
(PDF)

S7 Fig Examples of TP53 R273H and KRAS G12S castPCR™ and TaqMan® assays. Two-plex plots obtained from a single dPCR analysis using KRAS G12S castPCR™ assay (panels A and B) and TaqMan® assay (panels C and D), and TP53 R273H castPCR™ assay (panels E and F) and TaqMan® assay (panels G and H). As negative and positive controls, fragmented human wild-type only genomic DNA (A, C, E, G) and DNA from mutated cell lines (B, D, F, H) have been used. In both assays, final concentration of probes was of 1X, except for the VIC-labeled probe for TP53 Reference in castPCR™ assay which was used at a 0.5X final concentration (for TaqMan® assays, 0.8 μM of primers and 0.2 μM of probes). In the lower tables, droplets counts from experiments are listed. Input ng represents the amount of DNA used in dPCR, previously estimated by Qubit® 2.0 Fluorometer. *Reference*, *wild-type + mutant DNA; A*.*U*, *arbitrary units; gDNA*, *genomic DNA*.
(PDF)

S8 Fig Table showing events counts of mutation titration series. Mean of two or three replicates is shown. Expected percentage of mutations has been evaluated from the heterozygosity percentage of cell line obtained with each probe. Average and standard deviation for the different replicates are shown, both for WT and MUT-DNA containing droplets.
(PDF)

S9 Fig Controls scatter plots for the four-plex panel for the most common *EGFR* mutations. As control for the four-plex panel, the mutation mix has been used on different pools of DNA missing one of two cell lines (A and B panels). In the table, event counts from the single experiments are listed (input ng represents the amount of DNA used in dPCR, previously estimated by Qubit® 2.0 Fluorometer). *A*.*U*, *arbitrary units; Ctrl*, *control; WT*, *wild-type; S*, *sensitivity mutation; R*, *resistance mutation*.
(PDF)

S10 Fig False-positive evaluation in negative controls (human wild-type genomic DNA) for multiplex assays. In order to assess the false-positive (FP) events detected in negative control samples, we analyzed by dPCR a collection of human wild-type only samples (genomic DNA, refer to S1 Fig for details) with the multiplex EGFR tests previously described. We used two different amounts of DNA input (20 and 60 ng, depicted by circles and squares respectively). The right table shows the LOB and LOD estimation for all assays (refer to [33] for precise *formula*), calculated from the λ_FP_ of each test (where λ_FP_ is given by the mean number of FP obtained in all experiments realized with 20 ng input DNA). *N°*, *number; FP*, *false-positive; LOB*, *Limit of Blank; LOD*, *Limit of Detection*.
(PDF)

S11 Fig Comparison of the different assays performed using quantitative and digital PCR. Delta-CT values corresponding to the detection of mutant alleles (qPCR) or fraction of mutant DNA obtained by digital PCR are shown for each EGFR targeted mutation (A. p.L858R; B. p.L861Q; C. Del19; D. p.T790M). Results from healthy controls DNA and from lung cancer patients DNA are listed. Moreover, for each patient bearing a specific mutation, multiplex panel analysis is shown. *WT*, *wild-type; AF*, *allelic frequency; F*, *female; F-NS*, *non smoker female; M*, *male; M*, *non smoker male*.
(PDF)
