# Supplementary material for: Multiplex Detection of Rare Mutations by Picoliter Droplet Based Digital PCR: Sensitivity and Specificity Considerations
Source: PLoS One. 2016 Jul 14;11(7):e0159094. doi: 10.1371/journal.pone.0159094 (PMC4945036; doi:10.1371/journal.pone.0159094)
Supplement: S1 Fig — For p.L861Q EGFR assay, DNA from tumor collection available in the laboratory has been used (refer to Methods part). For p.R213* (637C>T) TP53 mutation, DNA was directly purchased from Cell Lines Service (CLS) Company. Horizon DiagnosticsTM cfDNA, Multiplex FFPE and Multiplex gDNA Reference Standards have also been tested. (PDF) [file pone.0159094.s001.pdf]

DNA origin	Cancer type	Mutation(s)	Genotype	Vendors (catalogue numbers)
genomic DNA	Human wild type DNA	-	-	Promega (G3041)
H1975	Non-small-cell lung carcinoma (NSCLC)	EGFR: p.L858R, p.T790M	Heterozygous	ATCC (CRL-5908)
H1650	Lung stage 3B,adenocarcinoma; bronchoalveolar carcinoma	EGFR: Del19	Heterozygous	ATCC (CRL-5883)
A427	Lung carcinoma	KRAS: p.G12D	Heterozygous	ATCC (HTB-53)
LS123	Colorectal adenocarcinoma	KRAS: p.G12S	Heterozygous	ATCC (CCL-255)
HT-29	Colorectal carcinoma	TP53: p.R273H	Homozygous	ATCC (HTB-38)
SW-684	Fibrosarcoma	TP53: p.R213*	Homozygous	CLS (300422GD1)
-	EGFR Gene-Specific Multiplex FFPE Reference Standard (5% AF)	EGFR: p.L861Q, ΔE746-A750, p.L858R, p.T790M, p.G719S	-	Horizon Diagnostics (HD850)
-	EGFR Gene-Specific Multiplex gDNA Reference Standard (12.5% AF)	EGFR: p.L861Q, ΔE746-A750, p.L858R, p.T790M	-	Horizon Diagnostics (HD802)
-	Multiplex I cfDNA Reference Standard Set (5-1-0.1% AF)	EGFR: p.L858R, ΔE746-A750, p.T790M, V769 - D770insASV	-	Horizon Diagnostics (HD780)

S1 FIG
