# Supplementary material for: Multiplex Detection of Rare Mutations by Picoliter Droplet Based Digital PCR: Sensitivity and Specificity Considerations
Source: PLoS One. 2016 Jul 14;11(7):e0159094. doi: 10.1371/journal.pone.0159094 (PMC4945036; doi:10.1371/journal.pone.0159094)
Supplement: S2 Fig — (PDF) [file pone.0159094.s002.pdf]

Reagents	Final concentration	Vendors (catalogue numbers)
TaqMan® Genotyping Master Mix	1X	Life Technologies (4371355)
dNTPs	1.2 mM	Life Technologies (R1121)
MgCl2	0.5 mM	Life Technologies (R0971)
Droplet Stabilizer	1X	RainDance Technologies (20-00803)
TaqMan® probes	0.2 or 0.4 µM	Life Technologies
Olinucleotide primers	0.8 or 1.6 µM	Life Technologies
castPCR™ probes	0.5, 1, 2 or 3X	Life Technologies
ZEN probes	0.2, 0.4 or 0.8 µM	IDT
Olinucleotide primers	0.4, 0.8 or 1.2 µM	IDT
Tris-HCl pH8 10 mM	to volume	Life Technologies (AM9855G)
DNA (WT or mutant)	≈ 300 to 800 copies/µL	Promega, ATCC, CLS or patient's DNA (see S1 Fig)

S2 FIG
