# Supplementary material for: Multiplex Detection of Rare Mutations by Picoliter Droplet Based Digital PCR: Sensitivity and Specificity Considerations
Source: PLoS One. 2016 Jul 14;11(7):e0159094. doi: 10.1371/journal.pone.0159094 (PMC4945036; doi:10.1371/journal.pone.0159094)
Supplement: S3 Fig — Main information about castPCR™, TaqMan® and ZEN™ assays are reported in the tables. The transcripts we refered to are NM_005228.3 for EGFR, NM_033360.2 for KRAS and NM_000546.4 for TP53 gene. *“MGBNFQ” refers to the minor groove binder non-fluorescent quencher; ** Proprietary information of Life Technologies-Thermo Fisher Scientific (sequences of probes and primers are not furnished). (PDF) [file pone.0159094.s003.pdf]

	castPCR™ probes					TaqMan® probes	ZEN™ probe
	**EGFR Ref**	**EGFR Del19**	**EGFR L858R**	**EGFR L861Q**	**EGFR T790M**	**EGFR L858R**	**EGFR T790M**
Assay name	EGFR_rf	EGFR_ex19dels_mu	EGFR_6224_mu	EGFR_6213_mu	EGFR_6240_mu	-	-
Nucleotide Variant	WT	19 deletions	c.2573T>G	c.2582T>A	c.2369C>T	c.2573T>G	c.2573T>G
Assay genome location	chr.7 55238406	chr.7 55242469-552424XX	chr.7 55259514-55259515	chr.7 55259524	chr.7 55249071	-	-
Assay gene location	intron 15	exon 19	exon 21	exon 21	exon 20	exon 21	exon 20
Forward Primer	**	**	**	**	**	5'-GCAGCATGTCAAGATCACAGATT-3'	5'-CCT CAC CTC CAC CGT GCA-3'
Reverse Primer	**	**	**	**	**	5'-CCTCCTTCTGCATGGTATTCTTTCT-3'	5'- AGG CAG CCG AAG GGC A-3'
WT probe	**	-	-	-		5'-/VIC/-AGTTTGGCCAGCCCAA-/MGBNFQ*/-3'	5'-/5TET/T+CA TC+A+C+GC/ZEN/A+GC TC/3IABkFQ/-3'
MUT probe	-	**	**	**	**	5'-/6FAM/-AGTTTGGCCCGCCCAA-/MGBNFQ*/-3'	5'-/56-FAM/T+CA TC+A+T+GC/ZEN/A+GC+TC/3IABkFQ/-3'
Amplicon (bp)	-	-	-	-	-	78	51
Catalog Number	4465807	4465805	4465804	4465804	4465804	custom	custom

	castPCR™ probes		TaqMan® probes	
	**KRAS Ref**	**KRAS G12S**	**KRAS G12D**	**KRAS G12S**
Assay name	KRAS_rf	KRAS_517_mu	-	-
Nucleotide Variant	WT	c.34G>A	c.35G>A	c.34G>A
Assay genome location	chr.12 25378771	chr.12 25398285	-	-
Assay gene location	intron 3	exon 2	exon 2	exon 2
Forward Primer	**	**	5'-AGGCCTGCTGAAAATGACTGAATAT-3'	5'-AGGCCTGCTGAAAATGACTGAATAT-3'
Reverse Primer	**	**	5'-GCTGTATCGTCAAGGCACTCTT-3'	5'-GCTGTATCGTCAAGGCACTCTT-3'
WT probe	**	-	5'-/VIC/-TTGGAGCTGGTGGCGT-/MGBNFQ*/-3'	5'-/VIC/-CTACGCCACCAGCTC-/MGBNFQ*/-3'
MUT probe	-	**	5'-/6FAM/-TGGAGCTGATGGCGT-/MGBNFQ*/-3'	5'-/6FAM/-CTACGCCACTAGCTC-/MGBNFQ*/-3'
Amplicon (bp)	-	-	80	80
Catalog Number	4465807	4465804	custom	custom

	castPCR™ probes			TaqMan® probe
	**TP53 Ref**	**p.R273H**	**p.R213***	**p.R273H**
Assay name	TP53_rf	TP53_10660_mu	TP53_10654_mu	-
Nucleotide Variant	WT	c.818G>A	c.637C>T	c.818G>A
Assay genome location	chr.17 7590792	chr.17 7577120	chr.17 7578212	-
Assay gene location	exon 1	exon 8	exon 6	exon 8
Forward Primer	**	**	**	5'-TGGTAATCTACTGGGACGGAACAGC-3'
Reverse Primer	**	**	**	5'-GGAGATTCTCTTCCTCTGTG-3'
WT probe	**	-	-	5'-/VIC/-ACAAACACGCACCTCA-/MGBNFQ*/-3'
MUT probe	-	**	**	5'-/6FAM/-ACAAACATGCACCTCA-/MGBNFQ*/-3'
Amplicon (bp)	-	-	-	86
Catalog Number	4465807	4465804	4465804	custom

S3 FIG
