# Supplementary material for: Multiplex Detection of Rare Mutations by Picoliter Droplet Based Digital PCR: Sensitivity and Specificity Considerations
Source: PLoS One. 2016 Jul 14;11(7):e0159094. doi: 10.1371/journal.pone.0159094 (PMC4945036; doi:10.1371/journal.pone.0159094)
Supplement: S4 Fig — CastPCR™ technology (upper panel) permits specific amplification of one allele type (bringing the mutation) while an MGB blocker suppresses the wild-type sequence (if present) at the mutation site. As reference, a TaqMan® system permits the amplification of wild-type DNA further away from the targeted mutation (e.g. about 20.000 nucleotides for EGFR probes) (refer to http://www.appliedbiosystems.com/absite/us/en/home/applications-technologies/real-time-pcr/castpcr.printable.html for more information). The TaqMan® technology (lower left figure) permits the detection of the mutation by specific match of fluorescent probe with the sequence in which the mutation resides. ZEN™ Internal Quencher (lower rigth figure) system represents an original modification developed by IDT which helps in lowering background and increasing signal than traditional methods (refer to http://eu.idtdna.com/pages/products/gene-expression/custom-qpcr-probes for further information). (PDF) [file pone.0159094.s004.pdf]

Competitive Allele-Specific TaqMan® PCR (castPCR™) system

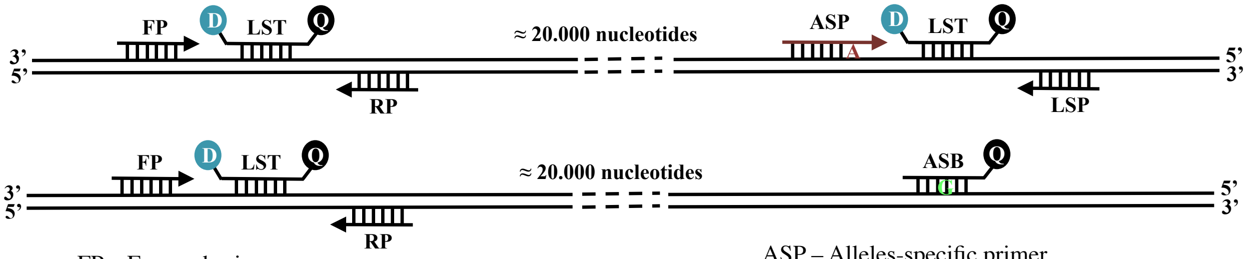

FP – Forward primer
RP – Reverse Primer
LST – Locus-specific TaqMan® probe

ASP – Alleles-specific primer
ASB – Alleles-specific blocker (MGB)
LST – Locus-specific TaqMan® probe
LSP – Locus-specific primer

TaqMan® system

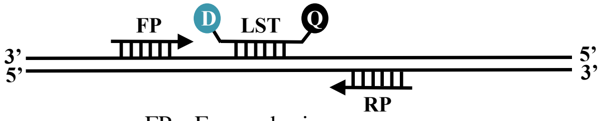

FP – Forward primer
RP – Reverse Primer
LST – Locus-specific TaqMan® probe

ZEN™ Internal Quencher system

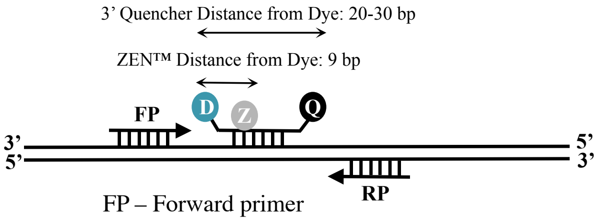

FP – Forward primer
RP – Reverse Primer

S4 FIG
