# Supplementary material for: Multiplex Detection of Rare Mutations by Picoliter Droplet Based Digital PCR: Sensitivity and Specificity Considerations
Source: PLoS One. 2016 Jul 14;11(7):e0159094. doi: 10.1371/journal.pone.0159094 (PMC4945036; doi:10.1371/journal.pone.0159094)
Supplement: S5 Fig — * For KRAS TaqMan® probes, an annealing temperature of 64°C has been used. (PDF) [file pone.0159094.s005.pdf]

	castPCR™ probes				TaqMan® probes				ZEN™ probes			
	**Temp (C°)**	**Hold**	**Rampe Rate (°C/s)**	**Cycles**	**Temp (C°)**	**Hold**	**Rampe Rate (°C/s)**	**Cycles**	**Temp (C°)**	**Hold**	**Rampe Rate (°C/s)**	**Cycles**
Pre-heating	-	-	-	-	50	2 min	0.6	1	-	-	-	-
Polymerase activation	95	10 min	0.6	1	95	10 min	0.6	1	95	10 min	0.6	1
Denaturation	92	15 s	0.6	5	95	15 s	0.6	45	95	15 s	0.6	45
Annealing & Extension	58	1 min	0.6		60*	1 min	0.6		58	15 s	0.6	
Denaturation	92	15 s	0.6	40	-	-	-	-	60	45 s	0.6	
Annealing & Extension	60	1 min	0.6		-	-	-	-	-	-	-	-
Incubation	98	10 min	0.6	1	98	10 min	0.6	1	98	10 min	0.6	1
Cooling	10	10 min	-	1	10	10 min	-	1	10	10 min	-	1

S5 FIG
