# Supplementary material for: Multiplex Detection of Rare Mutations by Picoliter Droplet Based Digital PCR: Sensitivity and Specificity Considerations
Source: PLoS One. 2016 Jul 14;11(7):e0159094. doi: 10.1371/journal.pone.0159094 (PMC4945036; doi:10.1371/journal.pone.0159094)
Supplement: S6 Fig — The amount of probes and primers used for duplex, triplex and quadruplex assays for the three targeted genes is shown. Since castPCR™ assays are selt as read-to-use by Life Technologies-Thermo Fisher Scientific company, we could not specifiy the final concentration (expressed in μM). (PDF) [file pone.0159094.s006.pdf]

Targeted mutation	2-plex assay		
	castPCR™ probes	TaqMan® probes	ZEN™ probe
EGFR L858R	1X VIC labeled probe for EGFR Reference<br>1X 6-FAM labeled probe for EGFR mutation	0.2 µM of of VIC and 6-FAM labeled probes<br>0.8 µM of primers	-<br>-
EGFR L861Q	1X VIC labeled probe for EGFR Reference<br>1X 6-FAM labeled probe for EGFR mutation	-<br>-	-<br>-
EGFR Del19	1X VIC labeled probe for EGFR Reference<br>1X 6-FAM labeled probe for EGFR mutation	-<br>-	-<br>-
EGFR T790M	1X VIC labeled probe for EGFR Reference<br>1X 6-FAM labeled probe for EGFR mutation	-<br>-	0.2 µM of of VIC and 6-FAM labeled probes<br>0.4 µM of primers

Targeted mutation	3-plex assay		
	castPCR™ probes	TaqMan® probes	ZEN™ probe
EGFR L858R	2X VIC labeled probe for EGFR mutation<br>2X 6-FAM labeled probe for EGFR mutation	-<br>-	-<br>-
EGFR L861Q	2X VIC labeled probe for EGFR mutation<br>2X 6-FAM labeled probe for EGFR mutation	-<br>-	-<br>-
EGFR Del19	-<br>0.5X 6-FAM labeled probe for EGFR mutation	-<br>-	-<br>-
EGFR T790M	-<br>-	-<br>-	0.2 µM of of VIC and 6-FAM labeled probes<br>0.4 µM of primers

Targeted mutation	4-plex assay		
	castPCR™ probes	TaqMan® probes	ZEN™ probe
EGFR L858R	1X VIC labeled probe for EGFR mutation<br>1X 6-FAM labeled probe for EGFR mutation	-<br>-	-<br>-
EGFR L861Q	-<br>-	-<br>-	-<br>-
EGFR Del19	-<br>0.5X 6-FAM labeled probe for EGFR mutation	-<br>-	-<br>-
EGFR T790M	-<br>-	-<br>-	0.2 µM of of VIC and 6-FAM labeled probes<br>0.4 µM of primers

Targeted mutation	2-plex assay	
	castPCR™ probes	TaqMan® probes
KRAS G12S/G12D	1X VIC labeled probe for KRAS Reference<br>1X 6-FAM labeled probe for KRAS mutation	0.2 µM of of VIC and 6-FAM labeled probes<br>0.8 µM of primers

Targeted mutation	2-plex assay	
	castPCR™ probes	TaqMan® probes
TP53 R213*/R273H	0.5X VIC labeled probe for TP53 Reference<br>1X 6-FAM labeled probe for TP53 mutation	0.2 µM of of VIC and 6-FAM labeled probes<br>0.8 µM of primers

S6 FIG
