# Supplementary material for: Multiplex Detection of Rare Mutations by Picoliter Droplet Based Digital PCR: Sensitivity and Specificity Considerations
Source: PLoS One. 2016 Jul 14;11(7):e0159094. doi: 10.1371/journal.pone.0159094 (PMC4945036; doi:10.1371/journal.pone.0159094)
Supplement: S7 Fig — Two-plex plots obtained from a single dPCR analysis using KRAS G12S castPCR™ assay (panels A and B) and TaqMan® assay (panels C and D), and TP53 R273H castPCR™ assay (panels E and F) and TaqMan® assay (panels G and H). As negative and positive controls, fragmented human wild-type only genomic DNA (A, C, E, G) and DNA from mutated cell lines (B, D, F, H) have been used. In both assays, final concentration of probes was of 1X, except for the VIC-labeled probe for TP53 Reference in castPCR™ assay which was used at a 0.5X final concentration (for TaqMan® assays, 0.8 μM of primers and 0.2 μM of probes). In the lower tables, droplets counts from experiments are listed. Input ng represents the amount of DNA used in dPCR, previously estimated by Qubit® 2.0 Fluorometer. Reference, wild-type + mutant DNA; A.U, arbitrary units; gDNA, genomic DNA. (PDF) [file pone.0159094.s007.pdf]

A. KRAS G12S castPCR™ assay on normal gDNA (WT only)

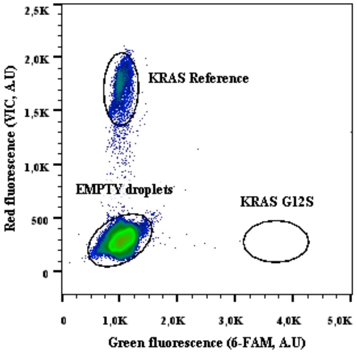

B. KRAS G12S castPCR™ assay on LS123 gDNA

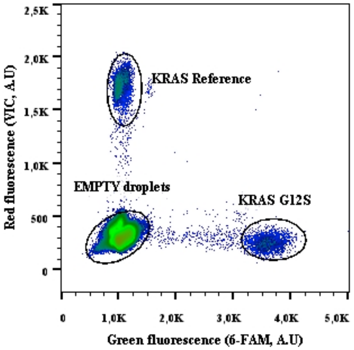

KRAS G12S castPCR™ probe		
	genomic DNA	LS123 cell line DNA
Wild-type DNA-containing droplets	5916	4832
Mutated DNA-containing droplets	0	2612
Input ng	20	20
% of mutated DNA	0%	54%

E. TP53 R273H castPCR™ assay on normal gDNA (WT only)

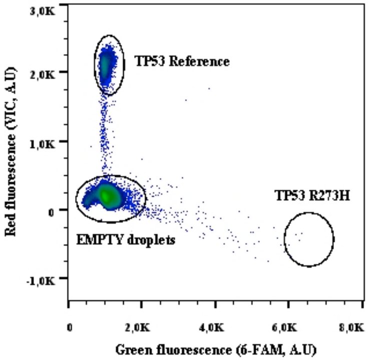

F. TP53 R273H castPCR™ assay on HT29 gDNA

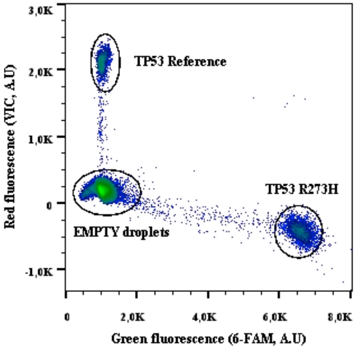

TP53 R273H castPCR™ probe		
	genomic DNA	HT29 cell line DNA
Wild-type DNA-containing droplets	8681	4332
Mutated DNA-containing droplets	6	4175
Input ng	20	20
% of mutated DNA	0%	96%

C. KRAS G12S TaqMan® assay on normal gDNA (WT only)

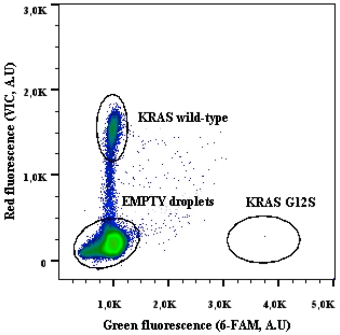

D. KRAS G12S TaqMan® assay on LS123 gDNA

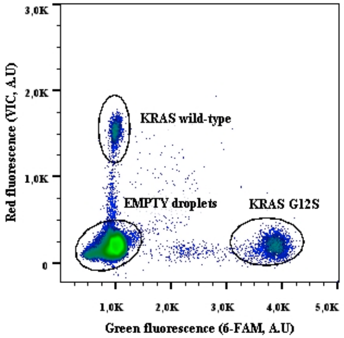

KRAS G12S TaqMan® probe		
	genomic DNA	LS123 cell line DNA
Wild-type DNA-containing droplets	8102	2241
Mutated DNA-containing droplets	0	4165
Input ng	20	20
% of mutated DNA	0%	65%

G. TP53 R273H TaqMan® assay on normal gDNA (WT only)

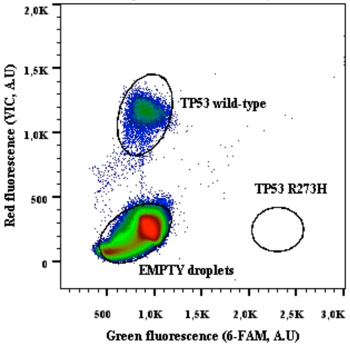

H. TP53 R273H TaqMan® assay on HT29 gDNA

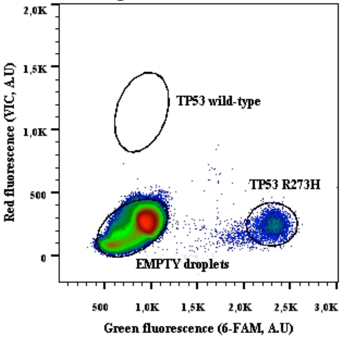

TP53 R273H TaqMan® probe		
	genomic DNA	HT29 cell line DNA
Wild-type DNA-containing droplets	9487	0
Mutated DNA-containing droplets	0	3822
Input ng	20	20
% of mutated DNA	0%	100%

S7 FIG
