# Supplementary material for: Multiplex Detection of Rare Mutations by Picoliter Droplet Based Digital PCR: Sensitivity and Specificity Considerations
Source: PLoS One. 2016 Jul 14;11(7):e0159094. doi: 10.1371/journal.pone.0159094 (PMC4945036; doi:10.1371/journal.pone.0159094)
Supplement: S8 Fig — Mean of two or three replicates is shown. Expected percentage of mutations has been evaluated from the heterozygosity percentage of cell line obtained with each probe. Average and standard deviation for the different replicates are shown, both for WT and MUT-DNA containing droplets. (PDF) [file pone.0159094.s008.pdf]

EGFR L858R castPCR™ probe

	0%		≈0.0090%		≈0.090%		≈0.90%		≈9.0%	
	Average	*Standard deviation*	*Average*	*Standard deviation*	*Average*	*Standard deviation*	*Average*	*Standard deviation*	*Average*	*Standard deviation*
Wild-type DNA-containing droplets	17038	261	52947	3523	15288	644	16080	727	17199	958
Mutated DNA-containing droplets	0	0	6	1	14	3	181	41	1965	195
% of mutation	0,000%		0,0112%		0,094%		1,12%		11,4%	

EGFR L858R TaqMan® probe

	0%		≈0.0075%		≈0.075%		≈0.75%		≈7.5%	
	Average	*Standard deviation*	*Average*	*Standard deviation*	*Average*	*Standard deviation*	*Average*	*Standard deviation*	*Average*	*Standard deviation*
Wild-type DNA-containing droplets	15835	4147	62290	4804	11758	9926	16501	3095	15077	3182
Mutated DNA-containing droplets	0	0	5	2	8	6	180	25	1533	301
% of mutation	0,000%		0,0073%		0,071%		1,08%		9,2%	

EGFR L861Q castPCR™ probe

	0%		≈0.0100%		≈0.100%		≈1.00%		≈10.0%	
	Average	*Standard deviation*	*Average*	*Standard deviation*	*Average*	*Standard deviation*	*Average*	*Standard deviation*	*Average*	*Standard deviation*
Wild-type DNA-containing droplets	17368	206	52486	2790	16031	1046	15880	650	17602	1073
Mutated DNA-containing droplets	1	1	3	1	17	6	204	40	204	40
% of mutation	0,004%		0,0066%		0,108%		1,28%		1,2%	

EGFR Del19 castPCR™ probe

	0%		≈0.0088%		≈0.088%		≈0.88%		≈8.8%	
	Average	*Standard deviation*	*Average*	*Standard deviation*	*Average*	*Standard deviation*	*Average*	*Standard deviation*	*Average*	*Standard deviation*
Wild-type DNA-containing droplets	17399	146	54062	3043	15652	1080	15349	816	16594	661
Mutated DNA-containing droplets	1	2	32	4	54	15	243	12	2061	172
% of mutation	0,007%		0,0599%		0,346%		1,58%		12,4%	

EGFR T790M castPCR™ probe

	0%		≈0.0060%		≈0.060%		≈0.60%		≈6.0%	
	Average	*Standard deviation*	*Average*	*Standard deviation*	*Average*	*Standard deviation*	*Average*	*Standard deviation*	*Average*	*Standard deviation*
Wild-type DNA-containing droplets	17192	715	53971	3225	15739	604	16223	653	17165	1089
Mutated DNA-containing droplets	21	8	158	40	48	12	172	41	1605	197
% of mutation	0,124%		0,2922%		0,303%		1,1%		9,3%	

EGFR T790M Prime-time LNA-ZEN probe

	0%		≈0.0072%		≈0.072%		≈0.72%		≈7.2%	
	Average	*Standard deviation*	*Average*	*Standard deviation*	*Average*	*Standard deviation*	*Average*	*Standard deviation*	*Average*	*Standard deviation*
Wild-type DNA-containing droplets	20159	2497	74177	1975	18288	2417	18751	1255	17797	2380
Mutated DNA-containing droplets	1	0	9	2	17	7	192	20	1952	140
% of mutation	0,006%		0,0123%		0,092%		1,0%		9,9%	

KRAS G12S castPCR™ probe

	0%		≈0.0063%		≈0.063%		≈0.63%		≈6.3%	
	Average	*Standard deviation*	*Average*	*Standard deviation*	*Average*	*Standard deviation*	*Average*	*Standard deviation*	*Average*	*Standard deviation*
Wild-type DNA-containing droplets	18433	1476	47020	13171	20110	1328	20208	1382	19282	3031
Mutated DNA-containing droplets	6	2	4	3	10	2	70	26	686	176
% of mutation	0,030%		0,0083%		0,051%		0,3%		3,6%	

KRAS G12S TaqMan® probe

	0%		≈0.0063%		≈0.063%		≈0.63%		≈6.3%	
	Average	*Standard deviation*	*Average*	*Standard deviation*	*Average*	*Standard deviation*	*Average*	*Standard deviation*	*Average*	*Standard deviation*
Wild-type DNA-containing droplets	18903	4065	58244	841	20657	2594	19942	2583	19634	2025
Mutated DNA-containing droplets	1	1	5	3	14	0	100	14	1085	110
% of mutation	0,003%		0,0094%		0,065%		0,5%		5,2%	

KRAS G12D TaqMan® probe

	0%		≈0.0060%		≈0.060%		≈0.60%		≈6.0%	
	Average	*Standard deviation*	*Average*	*Standard deviation*	*Average*	*Standard deviation*	*Average*	*Standard deviation*	*Average*	*Standard deviation*
Wild-type DNA-containing droplets	15292	6929	59512	429	21781	2136	21678	1609	17790	1963
Mutated DNA-containing droplets	0	0	7	1	9	1	80	9	893	49
% of mutation	0,000%		0,0112%		0,042%		0,4%		4,8%	

TP53 R273H castPCR™ probe

	0%		≈0.0100%		≈0.100%		≈1.00%		≈10.0%	
	Average	*Standard deviation*	*Average*	*Standard deviation*	*Average*	*Standard deviation*	*Average*	*Standard deviation*	*Average*	*Standard deviation*
Wild-type DNA-containing droplets	18023	967	52894	1886	16589	1387	18075	1082	19177	4637
Mutated DNA-containing droplets	39	13	114	23	55	15	217	16	1961	502
% of mutation	0,217%		0,2160%		0,333%		1,20%		10,2%	

TP53 R273H TaqMan® probe

	0%		≈0.0100%		≈0.100%		≈1.00%		≈10.0%	
	Average	*Standard deviation*	*Average*	*Standard deviation*	*Average*	*Standard deviation*	*Average*	*Standard deviation*	*Average*	*Standard deviation*
Wild-type DNA-containing droplets	19092	96	57622	4999	19993	2116	19914	1745	18245	2201,01902
Mutated DNA-containing droplets	0	0	7	1	17	9	182	19	1679	166,471272
% of mutation	0,000%		0,0122%		0,087%		0,91%		8,4%	

TP53 R213* castPCR™ probe

	0%		≈0.0100%		≈0.100%		≈1.00%		≈10.0%	
	Average	*Standard deviation*	*Average*	*Standard deviation*	*Average*	*Standard deviation*	*Average*	*Standard deviation*	*Average*	*Standard deviation*
Wild-type DNA-containing droplets	16517	383	54824	1176	16147	272	16511	688	16866	100,916134
Mutated DNA-containing droplets	34	0	99	33	48	6	230	33	2073	7418,28%
% of mutation	0,206%		0,1815%		0,299%		1,39%		12,3%	

S8 FIG
