# Supplementary material for: Multiplex Detection of Rare Mutations by Picoliter Droplet Based Digital PCR: Sensitivity and Specificity Considerations
Source: PLoS One. 2016 Jul 14;11(7):e0159094. doi: 10.1371/journal.pone.0159094 (PMC4945036; doi:10.1371/journal.pone.0159094)
Supplement: S9 Fig — As control for the four-plex panel, the mutation mix has been used on different pools of DNA missing one of two cell lines (A and B panels). In the table, event counts from the single experiments are listed (input ng represents the amount of DNA used in dPCR, previously estimated by Qubit® 2.0 Fluorometer). A.U, arbitrary units; Ctrl, control; WT, wild-type; S, sensitivity mutation; R, resistance mutation. (PDF) [file pone.0159094.s009.pdf]

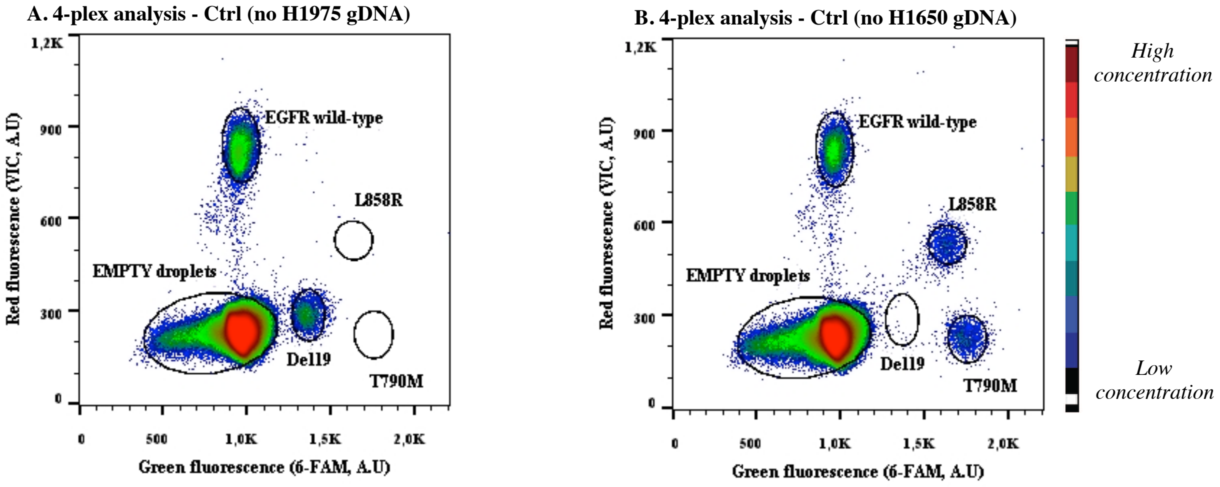

EGFR L858R-Del19 castPCR™ probes; EGFR T790M ZEN™ probe		
	A. 4-plex analysis - Ctrl (no H1975)	B. 4-plex analysis - Ctrl (no H1650)
	WT-L858R(S1)-Del19(S3)-T790M(R)	
Wild-type DNA-containing droplets (WT)	13060	6756
Mutated DNA-containing droplets (S1)	0	1165
Mutated DNA-containing droplets (S3)	3153	16
Mutated DNA-containing droplets (R)	0	1470
Input ng	7	7
% of mutation (S1)	0%	14%
% of mutation (S3)	24%	0%
% of mutation (R)	0%	18%

S9FIG
