# Supplementary material for: Multiplex Detection of Rare Mutations by Picoliter Droplet Based Digital PCR: Sensitivity and Specificity Considerations
Source: PLoS One. 2016 Jul 14;11(7):e0159094. doi: 10.1371/journal.pone.0159094 (PMC4945036; doi:10.1371/journal.pone.0159094)
Supplement: S10 Fig — In order to assess the false-positive (FP) events detected in negative control samples, we analyzed by dPCR a collection of human wild-type only samples (genomic DNA, refer to S1 Fig for details) with the multiplex EGFR tests previously described. We used two different amounts of DNA input (20 and 60 ng, depicted by circles and squares respectively). The right table shows the LOB and LOD estimation for all assays (refer to [33] for precise formula), calculated from the λFP of each test (where λFP is given by the mean number of FP obtained in all experiments realized with 20 ng input DNA). N°, number; FP, false-positive; LOB, Limit of Blank; LOD, Limit of Detection. (PDF) [file pone.0159094.s010.pdf]

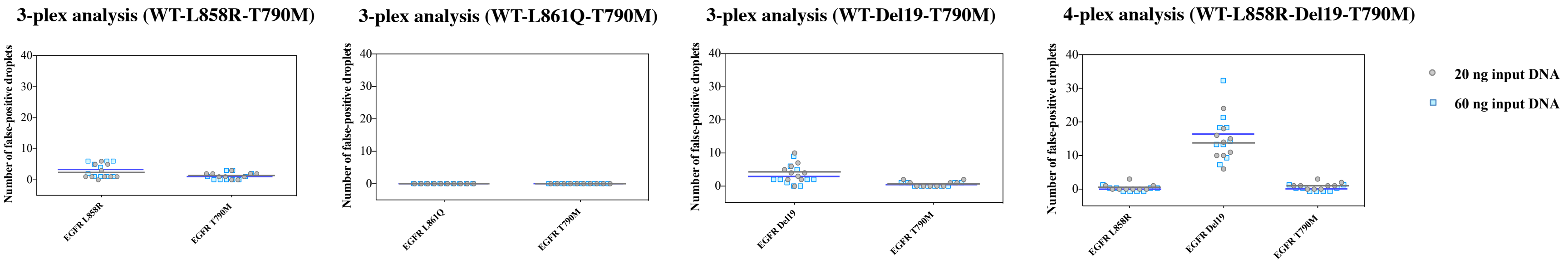

								MUT S1-DNA containing droplets		MUT R-DNA containing droplets		WT-DNA containing droplets	
	**N° of replicates**	**λ_{FP} S1**	**λ_{FP} R**	**LOB S1**	**LOB R**	**LOD S1**	**LOD R**	*Average*	*Standard deviation*	*Average*	*Standard deviation*	*Average*	*Standard deviation*
EGFR L858R-T790M	10	2,4	1,4	6	4	11	9	2	2	1	1	4642	132
EGFR L861Q-T790M	10	0	0	0	0	3	3	0	0	0	0	7366	287
EGFR Del19-T790M	10	4,3	0,7	9	3	15	7	4	3	1	1	7198	320

											MUT L858R-DNA containing droplets		MUT Del19-DNA containing droplets		MUT T790M-DNA containing droplets		WT-DNA containing droplets	
	**N° of replicates**	**λ_{FP} L858R**	**λ_{FP} Del19**	**λ_{FP} T790M**	**LOB L858R**	**LOB Del19**	**LOB T790M**	**LOD L858R**	**LOD Del19**	**LOD T790M**	*Average*	*Standard deviation*	*Average*	*Standard deviation*	*Average*	*Standard deviation*	*Average*	*Standard deviation*
EGFR L858R-Del19-T790M	9	0,56	13,78	1	3	21	3	7	30	8	1	1	14	5	1	1	6964	302

S10 FIG
