# Supplementary material for: Multiplex Detection of Rare Mutations by Picoliter Droplet Based Digital PCR: Sensitivity and Specificity Considerations
Source: PLoS One. 2016 Jul 14;11(7):e0159094. doi: 10.1371/journal.pone.0159094 (PMC4945036; doi:10.1371/journal.pone.0159094)
Supplement: S11 Fig — Delta-CT values corresponding to the detection of mutant alleles (qPCR) or fraction of mutant DNA obtained by digital PCR are shown for each EGFR targeted mutation (A. p.L858R; B. p.L861Q; C. Del19; D. p.T790M). Results from healthy controls DNA and from lung cancer patients DNA are listed. Moreover, for each patient bearing a specific mutation, multiplex panel analysis is shown. WT, wild-type; AF, allelic frequency; F, female; F-NS, non smoker female; M, male; M, non smoker male. (PDF) [file pone.0159094.s011.pdf]

A. qPCR and dPCR analysis of DNA extracted from healthy subjects and lung cancer patients bearing an EGFR p.L858R initial mutation in the tumor

		qPCR	dPCR					
		two-plex	two-plex WT-L858R	three-plex WT-L858R-T790M		four-plex WT-L858R-Del19-T790M		
		ΔCt value	AF % L858R	AF % L858R	AF % T790M	AF % L858R	AF % Del19	AF % T790M
Normal controls	F 06	ND	0.00%	0.000%	0.000%	0.152% *	2.225% *	0.153% *
	F-NS-03	ND	0.00%	0.423% *	0.000%	0.000%	0.000%	0.000%
	M 01	ND	0.00%	0.303% *	0.000%	0.439% *	1.042% *	0.000%
	M-NS-01	ND	0.29% *	0.000%	0.000%	0.174% *	0.000%	0.000%
Cancer patients	Patient n°1	6.070	2.59%	2.922%	0.000%	0.573%	0.000%	0.000%
	Patient n°2	ND	1.29%	0.825%	0.000%	0.956%	0.000%	0.000%

B. qPCR and dPCR analysis of DNA extracted from healthy subjects and lung cancer patients bearing an EGFR p.L861Q initial mutation in the tumor

		qPCR	dPCR		
		two-plex	two-plex WT-L861Q	three-plex WT-L861Q-T790M	
		ΔCt value	AF % L861Q	AF % L861Q	AF % T790M
Normal controls	F 06	ND	0.00%	0.000%	0.714% *
	F-NS-03	ND	0.00%	0.775% *	0.000%
	M 01	ND	0.00%	0.244% *	0.122% *
	M-NS-01	ND	0.00%	0.000%	0.000%
Cancer patients	Patient n°3	ND	23.44%	18.136%	0.000%
	Patient n°4	ND	18.54%	13.403%	0.066% *

C. qPCR and dPCR analysis of DNA extracted from healthy subjects and lung cancer patients bearing an EGFR Del19 initial mutation in the tumor

		qPCR	dPCR					
		two-plex	two-plex WT-Del19	three-plex WT-Del19-T790M		four-plex WT-L858R-Del19-T790M		
		ΔCt value	AF % Del19	AF % Del19	AF % T790M	AF % L858R	AF % Del19	AF % T790M
Normal controls	F 06	ND	0.00%	0.000%	0.000%	0.152% *	2.225% *	0.153% *
	F-NS-03	ND	0.00%	0.000%	0.000%	0.000%	0.000%	0.000%
	M 01	ND	0.00%	0.000%	0.000%	0.439% *	1.042% *	0.000%
	M-NS-01	ND	0.00%	0.000%	0.000%	0.174% *	0.000%	0.000%
Cancer patients	Patient n°5	7,76	0.53%	0.431%	0.000%	0.000%	2.969%	0.000%
	Patient n°6	ND	1.38%	0.342% *	0.000%	0.000%	0.112% *	0.000%

D. qPCR and dPCR analysis of DNA extracted from healthy subjects and lung cancer patients bearing an EGFR p.T790M initial mutation in the tumor

		qPCR	dPCR					
		two-plex	two-plex WT-T790M	three-plex WT-Del19-T790M		four-plex WT-L858R-Del19-T790M		
		ΔCt value	AF % T790M	AF % Del19	AF % T790M	AF % L858R	AF % Del19	AF % T790M
Normal controls	F 06	0.09	0.00%	0.000%	0.000%	0.152% *	2.225% *	0.153% *
	F-NS-03	ND	0.00%	0.000%	0.000%	0.000%	0.000%	0.000%
	M 01	ND	0.00%	0.000%	0.000%	0.439% *	1.042% *	0.000%
	M-NS-01	ND	0.00%	0.000%	0.000%	0.174% *	0.000%	0.000%
Cancer patients	Patient n°7	7,77	5.17%	0.000%	4.598%	5.691%	0.000%	3.570%
	Patient n°8	15,59	8.82%	0.000%	3.150%	0.000%	0.000%	1.027%

* Events in mutated DNA-containing cluster are lower than LOD value specific of the test.

S11 FIG
